# The ESCRT Machinery Is Recruited by the Viral BFRF1 Protein to the Nucleus-Associated Membrane for the Maturation of Epstein-Barr Virus

**DOI:** 10.1371/journal.ppat.1002904

**Published:** 2012-09-06

**Authors:** Chung-Pei Lee, Po-Ting Liu, Hsiu-Ni Kung, Mei-Tzu Su, Huey-Huey Chua, Yu-Hsin Chang, Chou-Wei Chang, Ching-Hwa Tsai, Fu-Tong Liu, Mei-Ru Chen

**Affiliations:** 1 Graduate Institute and Department of Microbiology, College of Medicine, National Taiwan University, Taipei, Taiwan; 2 Center of General Education, National Taipei University of Nursing and Health Sciences, Taipei, Taiwan; 3 Department of Anatomy and Cell Biology, College of Medicine, National Taiwan University, Taipei, Taiwan; 4 Institute of Biomedical Sciences, Academia Sinica, Taipei, Taiwan; 5 Department of Dermatology, School of Medicine, University of California, Davis, California, United States of America; University of California at Los Angeles, United States of America

## Abstract

The cellular endosomal sorting complex required for transport (ESCRT) machinery participates in membrane scission and cytoplasmic budding of many RNA viruses. Here, we found that expression of dominant negative ESCRT proteins caused a blockade of Epstein-Barr virus (EBV) release and retention of viral BFRF1 at the nuclear envelope. The ESCRT adaptor protein Alix was redistributed and partially colocalized with BFRF1 at the nuclear rim of virus replicating cells. Following transient transfection, BFRF1 associated with ESCRT proteins, reorganized the nuclear membrane and induced perinuclear vesicle formation. Multiple domains within BFRF1 mediated vesicle formation and Alix recruitment, whereas both Bro and PRR domains of Alix interacted with BFRF1. Inhibition of ESCRT machinery abolished BFRF1-induced vesicle formation, leading to the accumulation of viral DNA and capsid proteins in the nucleus of EBV-replicating cells. Overall, data here suggest that BFRF1 recruits the ESCRT components to modulate nuclear envelope for the nuclear egress of EBV.

## Author Summary

Herpesviruses are large DNA viruses associated with human and animal diseases. After viral DNA replication, the herpesviral nucleocapsids egress through the nuclear membrane for subsequent cytoplasmic virion maturation. However, the mechanism by which the virus regulates the nuclear membrane and cellular machinery involved in this process remained elusive. The cellular endosomal sorting complex required for transport (ESCRT) machinery is known to participate in the biogenesis of multivesicular bodies, cytokinesis and the release of enveloped viruses from cytoplasmic membranes. Here, we show that functional ESCRT machinery is required for the maturation of Epstein-Barr virus (EBV). ESCRT proteins are redistributed close to the nucleus-associated membrane through interaction with the viral BFRF1 protein, leading to vesicle formation and structural changes of the nuclear membrane. Remarkably, inhibition of ESCRT machinery abolishes BFRF1-induced vesicle formation, and leads to the accumulation of viral DNA and capsid proteins in the nucleus. Specific interactions between BFRF1 and Alix are required for BFRF1-derived vesicle formation and crucial for the nuclear egress of EBV.

## Introduction

The endosomal sorting complex required for transport (ESCRT) machinery is conserved evolutionarily and involved in catalyzing the scission of membrane necks in endosome sorting, biogenesis of multivesicular bodies (MVBs), cytokinesis and release of enveloped virions. In contrast to the cellular membrane-scission protein dynamin family, which cleaves membrane necks by constricting them from the outside, membrane scission mediated by the ESCRT machinery is from inside the neck (reviewed in [1], [2]). The ESCRT components (also known as class E proteins) consist of five multiprotein complexes, ESCRT-0, -I, -II, -III, Vps4 (vacuolar protein sorting-4) ATPase, and several ESCRT-associated proteins [3], [4]. ESCRT-0, -I, and -II are soluble complexes that shuttle between cytosolic and membrane-bound forms. These components sequentially coordinate together to bud the membrane and recruit ESCRT-III for the scission of membrane neck. ESCRT-III proteins belong to the Chmp family and are soluble monomers that assemble on membranes to form tight filamentary spirals and are released from the membranes at the final stage with other ESCRT proteins by the transient ATP-dependent reaction of Vps4. In addition to the regular composition, cellular ESCRT-I protein TSG101 (tumor susceptibility gene 101) alternatively activates the spiral assembly of ESCRT-III through bridging by the ESCRT associated protein apoptosis linked gene-2 interacting protein X (Alix) [5]. Because these class E proteins are recruited sequentially and assembled for their functions, the interaction-disrupted mutants of Alix and Chmps, as well as the ATPase activity defective Vps4 (e.g. Vps4A^E228Q^), are useful tools to investigate the involvement of the ESCRT machinery in various biological processes [6]–[9].

In addition to physiological functions, components of the ESCRT machinery are used by many enveloped viruses for budding and release from cells. By sequence comparison, late-budding (L) domains have been identified extensively in the structural proteins of these viruses by three conserved tetrapeptide motifs [Y(L)XXL, PT/SAP and PPXY] that mediate the recruitment and interaction of class E proteins to facilitate virus budding [10], [11]. Among the L domains, Y(L)XXL-, PT/SAP-, and PPXY-type motifs interact specifically with ESCRT associated Alix, TSG101 and Nedd4-like E3 ubiquitin ligases (e.g. Trp-Trp-domain-containing protein-1), respectively. Substitutions in the interacting motifs of the structural proteins also lead to defects in virion maturation and release [6]. The dynamics of ESCRT protein recruitment in retroviruses were found to be extremely transient (∼1–3 min) and sufficient for their functions on the membrane for virus release [12]. In contrast to enveloped RNA viruses, the contribution of ESCRT machinery to the maturation of enveloped herpesviruses remains to be explored.

Herpesviruses are large DNA viruses associated with human and animal diseases. After viral DNA replication, the newly synthesized genomes are packaged into pre-assembled intranuclear capsids. Based on the current envelopment-deenvelopment-reenvelopment model, large-sized herpesviral nucleocapsids (115–130 nm) begin budding through a transient envelopment process with the nuclear envelope. This is first mediated by the viral protein kinase and nuclear membrane associated proteins at the inner nuclear membrane (INM) for the local disassembly of compact nuclear lamina for primary envelopment. After release from the nuclear envelope derived structures, the nucleocapsids subsequently become associated with viral tegument proteins and glycoproteins at cytoplasmic apparatuses for final maturation of virions (reviewed in [13], [14]). So far, the involvement of ESCRT in virion release, and cytoplasmic reenvelopment of herpes simplex type 1 (HSV-1) and human cytomegalovirus (HCMV) have been characterized using dominant negative (DN) inhibitors of ESCRT and siRNA strategies [7]–[9], [15]. By immunofluorescence analysis, the cytoplasmic nucleocapsids and envelope components are associated with MVB and colocalized with endosomal markers in infected cells [16], [17]. As observed by electron microscopy (EM) analysis, HHV-6 also induces MVB formation and cytoplasmic egress through an exosomal release pathway [18], suggesting that herpesviruses use the ESCRT machinery for their membrane-dependent maturation in the cytoplasm. Knowledge regarding the nuclear egress of nucleocapsids has emerged only recently.

Several viral proteins have been characterized and shown to regulate the primary envelopment of herpesviruses from the nuclear membrane, in particular the herpesvirus conserved homologs of UL34 and UL31. The homologs of HSV-1 UL34 are type II integral membrane proteins that localize predominantly to the INM, outer nuclear membrane (ONM) and ER, whereas UL31 homologs are nuclear matrix-associated phosphoproteins [19]–[23]. The homolog pairs UL34/UL31 are conserved among herpesviruses and codependent for their localization to the nuclear rim. They also share the ability to interact with nuclear lamin proteins. Transient overexpression of the UL34/UL31 homologs of HSV-1 or HCMV induces subtle alterations of the nuclear lamina, which are distinguishable from the dramatic redistribution of lamin proteins in cells replicating the virus [24]–[26]. This suggests that the homologs of UL34 and UL31 potentially regulate the structure of the nuclear membrane, but coordination with other viral proteins is required for the fine-tuning of nuclear egress. In addition to viral products, the preformed UL34/UL31 homolog complexes in alpha- and beta-herpesviruses can recruit cellular factors, such as PKCs and lamin B receptor (LBR), and assemble into complexes to facilitate the nuclear egress of nucleocapsids [27]–[30]. However, the cellular factors contributing to the nuclear membrane budding (primary envelopment) of nucleocapsids remain elusive.

Epstein-Barr virus (EBV) is a gammaherpesvirus that infects most of the human population. During lytic infection, EBV encodes several gene products that modulate the cellular environment and facilitate virion maturation [14]. The study of the nuclear egress of EBV was hampered particularly by the lack of an efficient replication system in vitro. In previous studies, we found that EBV BGLF4 kinase regulates the structure of nuclear lamina to facilitate the initiation of nucleocapsid egress [31], [32]. In addition to BGLF4 kinase, the gene products of BFRF1 and BFLF2, the UL34 and UL31 positional homologs of HSV-1, were shown to regulate the primary egress of nucleocapsids [33]. By an unclear mechanism, transient expression of BFRF1 in 293 cells induces the formation of multiplied nuclear membranes and cytoplasmic cisternal membrane structures, suggesting the potential contribution of EBV BFRF1 to membrane modification during nuclear egress [33].

More than 30 years ago, a pioneering ultrastructural study found that EBV lytic replication in transformed B cells induced alterations of the nuclear membrane consisting of deep enfolding or multilayered structures, accompanied by some irregular vacuoles with electron dense material in the cytoplasm (Figure 3 in [34]). Recently, using advanced electron tomography, special nuclear invagination structures of both nuclear membranes, containing multiple viral nucleocapsids, also were seen in cells infected with the replication-efficient murine gammaherpesvirus 68 (MHV-68) [35]. In order to explore the cellular machineries involved in EBV maturation, we set out to study the contribution of the ESCRT machinery to the EBV maturation process.

## Results

### The ESCRT machinery regulates the virus maturation of EBV

**Figure 1:**
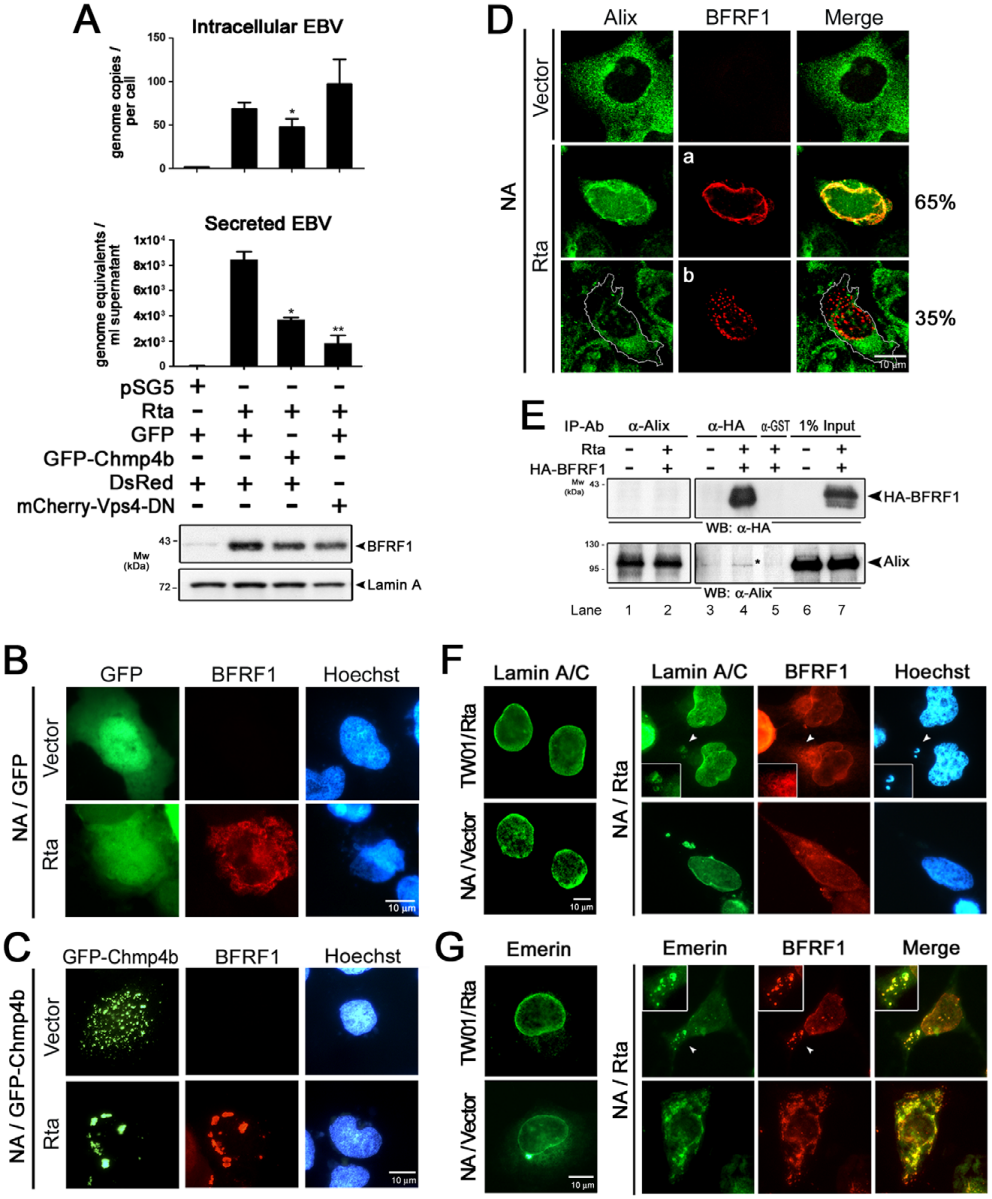
The ESCRT machinery contributes to EBV maturation and is redistributed to nucleus-associated compartments in EBV-reactivated NA cells. (**A**) EBV-positive NA cells were transfected with plasmids expressing GFP-Chmp4b, GFP (the GFP vector), mCherry-Vps4-DN or DsRed (the mCherry related vector) together with a plasmid expressing Rta or pSG5 vector control. The viral DNA content in the transfected cells and culture supernatant was harvested at 96 h post induction and subjected to qPCR analysis targeting the EBV DNA *Bam*HI W fragment. Results are means ± standard deviations from two separate transfections. Data are representatives of two independent experiments. The statistically significant differences between the Rta expression alone and coexpression of Rta and GFP-Chmp4b or mCherry-Vps4-DN are calculated by the paired Student's t-test and indicated at the top of the bars. *, P<0.05; **, P<0.01. The expressed EBV BFRF1 and cellular lamin A proteins also were detected by immunoblotting using anti-BFRF1 (E10) anti-lamin A/C. Expression of GFP-Chmp4b or Vps4-DN reduced the virion secretion. (**B and C**) To investigate the GFP-Chmp4b localization in EBV reactivated cells, slide-cultured NA cells were transfected with plasmids expressing GFP or GFP-Chmp4b, together with Rta expressing plasmid. At 48 h post transfection, cells were fixed by 4% paraformaldehyde and immuno-stained for BFRF1 (red). Cellular DNA was detected by Hoechst 33258 staining. (**D**) To detect Alix protein in EBV reactivated cells, slide-cultured NA cells were transfected with Rta expressing or vector plasmid. At 48 h post transfection, cells were then fixed, stained for Alix (green) and BFRF1 (red) and observed using confocal microscopy. The cell margin in b was detected by MetaMorph software and is indicated by a white outline. Two major types of BFRF1 distribution were observed in virus- reactivated cells: a, nuclear rim distribution (∼65%) and b, nuclear rim with cytoplasmic punctate distribution (∼35%). (**E**) Lysates from Rta-transfected NA cells were immunoprecipitated with antibody against Alix, HA or GST antibody as a negative control. The immunocomplexes were then resolved by 10% SDS-PAGE and immunoblotted with antibodies against HA or Alix. The coimmunoprecipitated proteins are indicated by “*”. (**F and G**) To observe the nuclear envelope structure in EBV-reactivated cells, NA or NPC-TW01 cells were transfected with a plasmid expressing Rta to induce virus lytic replication or vector plasmid. At 72 h post transfection, cells were fixed and immuno-stained for lamin A/C (green) and BFRF1 (red), or emerin (green) and BFRF1 (red) and stained with cellular DNA. Approximately 50∼100 cells with BFRF1 expression were counted for the appearance of cytoplasmic vesicle associated lamin A/C or emerin in each set of transfection, and all the experiments were repeated independently at least twice.

To determine whether the ESCRT machinery participates in EBV maturation, we used the EBV converted nasopharyngeal carcinoma (NPC) cell line NA, in which lytic replication can be induced by the expression of the viral immediate early protein Rta [36]. Among the ESCRT proteins, Chmp proteins mediate the formation of functional membrane scission complexes, and Vps4 controls the release of ESCRT complexes from membranes [2], [4], [37]. Inhibition of Chmps using GFP-tagged Chmp proteins, or Vps4 using catalytically inactivated Vps4-DN, traps the class E complexes and inhibits virion release by various viruses [8], [9], [12], [38]. Therefore, NA cells were transfected with Rta accompanied by the dominant negative (DN) forms of class E protein GFP-Chmp4b or catalytically inactivated Vps4A^E228Q^ mutant (Vps4-DN) plasmid. At 96 h post transfection, the cellular DNA and culture supernatant were harvested to test for EBV virion release. With about 50% transfection efficiency, we found the expression of GFP-Chmp4b or Vps4-DN reduced the amounts of secreted virions (∼44% and ∼22% of that of GFP transfected cells) as detected by quantitative PCR of the *Bam*HI W fragment of the EBV genome (Figure 1A). Even not very significant, there was a slight decrease of intracellular viral DNA in GFP-Chmp4 expressing cells and a slight increase of intracellular viral DNA in Vps4-DN expressing cells. In immunobloting, we found Rta transfection induced the expression of the EBV immediate-early protein Zta, early proteins BMRF1, BFRF1, BFLF2, and BGLF4, and major capsid structural proteins BcLF1 (VCA) and glycoprotein BLLF1 (gp350), and the expression was not affected by the coexpression of GFP-Chmp4b or Vps4-DN (Figure S1A). This indicates that inhibition of the ESCRT machinery interferes with EBV replication or virion release.

### EBV reactivation redistributes cellular ESCRT proteins, reorganizes the nuclear envelope and induces nuclear envelope-derived structure formation

To investigate the contribution of ESCRT proteins in EBV maturation, we examined the distribution of the ESCRT proteins in EBV replicating NA cells. Slide-cultured NA cells were transfected with plasmid expressing GFP or GFP-Chmp4b and Rta plasmid to induce the lytic cycle for 48 h. The subcellular localization of the viral nuclear envelope protein BFRF1 and the dominant negative GFP-Chmp4 were observed first. We found the BFRF1 protein showed a cytoplasmic punctate and perinuclear distribution in GFP-expressing Rta-induced NA cells (Figure 1B). With a moderate ability to block virion release in Figure 1A, the GFP-Chmp4b proteins were redistributed from a diffuse punctate pattern in vector plasmid transfected cells into clumping structures and colocalized with BFRF1 at the perinuclear region in cells replicating EBV (Figure 1C). Different from Chmp4b, Chmp1b is an accessory protein for MVB formation and dispensable for HIV budding [39], [40]. Here Chmp1b-GFP showed a homogenous distribution in vector transfected NA cells (Figure S1B, upper panels), whereas EBV reactivation by Rta transfection redistributed Chmp1b-GFP to cytoplasmic puncta, and colocalized with viral BFRF1 (Figure S1B, lower panels), suggesting Chmp1b is also involved in EBV reactivation-induced nuclear envelope modification.

Among the ESCRT proteins, bridging protein Alix is progressively recruited by membrane anchoring Gag for retroviral assembly [12], [39]. Chmps are subsequently activated by Alix in vivo [5]. Here we found in vector- or Rta-expressing EBV negative NPC-TW01 cells, the parental cell line of NA cells, Alix was localized in the cytoplasm in a diffuse pattern, similar to that of vector transfected cells (Figure S1C). In confocal images, EBV reactivation for 48 h apparently enhanced intranuclear and nuclear rim distribution of Alix (Figure 1D). The nuclear envelope morphology was affected by virus replication, and Alix was partially colocalized with viral BFRF1 protein at the nuclear rim (in 65% reactivated cells, merge). In cells with punctate BFRF1 containing structures, Alix showed nuclear rim or cytoplasmic distribution (in 35% reactivated cells), suggesting Alix may be released into cytoplasm after the formation of BFRF1 puncta. Because the ESCRT components function through a very transient interaction, it is reasonable that only small amounts of Alix were coimmunoprecipitated with HA-BFRF1 protein by anti-HA antibody in EBV reactivated NA cell lysates (Figure 1E, lane 4). Alix antibody precipitated the specific protein but without coimmunoprecipitating HA-BFRF1, possibly because of epitope blockage or instability of the immunocomplexes (Figure 1E, lane 2). Data here suggest Alix, as well as other ESCRT proteins, are redistributed to nuclear envelope associated compartments and associated with viral BFRF1 in cells replicating EBV.

While membrane modulating-ESCRT proteins were relocated near to the nucleus, the nuclear envelope structure of Rta-transfected cells was then monitored through immunostaining of viral and cellular nuclear membrane-associated proteins. We found that viral BFRF1 and BFLF2 were colocalized at the nuclear rim in EBV reactivated NA cells. Oriented punctate structures with perinuclear BFRF1 or BFLF2 staining were observed in about 90% of NA cells, especially in the concave region of the kidney shaped nucleus (Figure S1D, inset) or in the cytoplasm (Figure S1D, arrowhead). With Rta transfection, we found INM-associated lamin A/C and emerin were redistributed into the nuclear periphery or cytoplasmic region of the BFRF1-positive punctate structure in NA cells, but not in NPC-TW01 cells (Figure 1F and 1G). Cellular DNA was also detected in a proportion of cytoplasmic punctate structures with lamin and BFRF1 staining (in about 15–20% of cells expressing BFRF1, Figure 1F, arrowhead), suggesting that these cytoplasmic vesicles are derived from the reorganized nuclear membranes. Collectively, data here indicate that EBV reactivation redistributes ESCRT proteins, reorganizes the nuclear envelope and induces cytoplasmic nuclear envelope-derived structures.

### Expression of EBV BFRF1 reorganizes the nuclear membrane and induces vesicle formation from the nucleus-associated membrane

**Figure 2:**
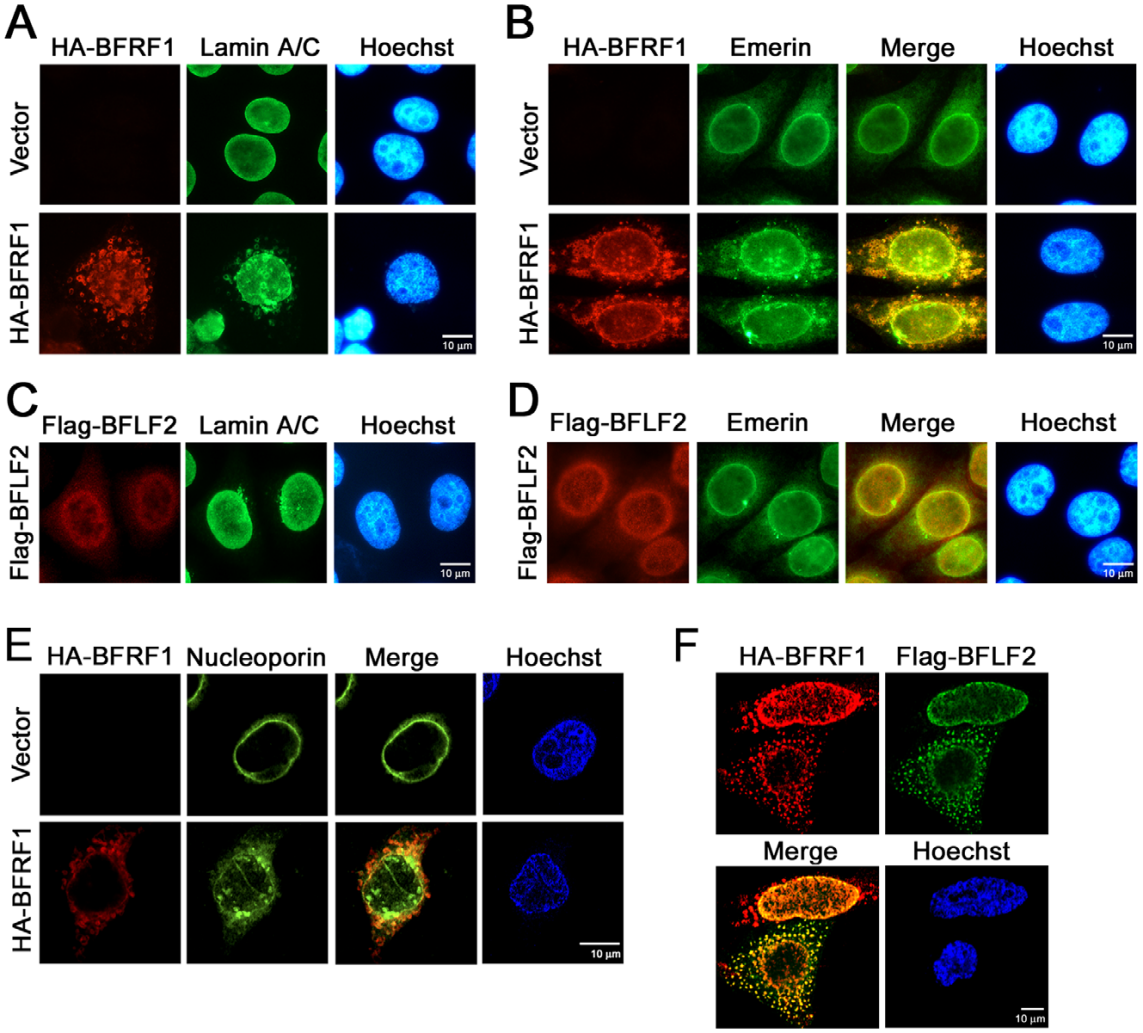
EBV BFRF1 reorganizes the nuclear membrane and induces vesicle formation from the altered membrane. (**A to D**) Slide-cultured HeLa cells were transfected with a plasmid expressing HA-BFRF1, Flag-BFLF2 or control vector for 24 h. The cells were fixed with 4% paraformaldehyde and stained for HA (A and B, red), Flag (C and D, red), lamin A/C (A and C, green) or emerin (B and D, green), and DNA. Approximately 50∼100 HA-BFRF1 expressing cells were counted to detect the cytoplasmic vesicle associated lamin (A and C) or emerin (B and D) in each set of transfections and all the experiments were repeated independently at least twice. (**E**) To investigate the effects of BFRF1 on nucleoporins distribution, HeLa cells were transfected with a plasmid expressing HA-BFRF1 or control vector for 24 h, stained for HA-BFRF1 (red) or FG-repeat containing nucleoporins (mAb414, green) and DNA, and analyzed by confocal microscopy. (**F**) HeLa cells were cotransfected with plasmid expressing HA-BFRF1 and Flag-BFLF2 for 24 h, fixed, stained for HA, Flag and DNA, and analyzed by confocal microscopy.

Because viral BFRF1 and BFLF2 colocalized with nuclear membrane proteins in cytoplasmic punctate structures, we determined whether BFRF1 or BFLF2 is responsible for the nuclear envelope reorganization using a transient expression system. Expression of HA-BFRF1 alone showed nuclear rim and cytoplasmic distribution in transfected HeLa cells (Figure 2A and 2B), whereas Flag-BFLF2 was predominantly distributed in the nucleus (Figure 2C and 2D). Different from the clumped perinuclear patterns or oriented punctate structures observed in cells replicating virus (Figure 1C, 1F, 1G and S1D), HA-BFRF1 induced dispersive vesicles in the cytoplasm of transfected cells (Figure 2A). Compared with the typical nuclear rim staining pattern of lamin A/C and emerin in vector transfected cells (Figure 2A and 2B, upper panel), cytoplasm-redistributed nuclear emerin with vesicle structure was found colocalized with HA-BFRF1 at the perinuclear region of 45∼55% cells with BFRF1 expression (Figure 2B, Merge). In contrast, the nuclear rim staining of lamin A/C and emerin was not affected by the expression of Flag-BFLF2 (Figure 2C and 2D). In addition to INM-anchoring proteins, we also detected the distribution of nuclear pore complexes by FG repeat-specific mAb414. FG repeat-containing nucleoporins showed a nuclear rim staining in control plasmid transfected cells (Figure 2E). Expression of HA-BFRF1 induced the redistribution of FG-repeat containing nucleoporins into perinuclear and intranuclear puncta, partially associated with perinuclear HA-BFRF1 containing vesicles.

Because BFRF1 and BFLF2 function together for the nuclear egress of EBV, the distribution of both proteins was detected in cotransfected HeLa cells (Figure 2F). Similar to cells expressing HA-BFRF1 alone, HA-BFRF1 distributed at the nuclear rim and in dispersive cytoplasmic vesicles in cells co-expressing HA-BFRF1 and Flag-BFLF2. Flag-BFLF2 either showed nuclear rim and cytoplasmic colocalization patterns with HA-BFRF1 or was expressed in the nucleus of co-transfected cells. This suggests that BFRF1 alone is capable of inducing nuclear membrane associated vesicles, whilst BFRF1 can further recruit the nuclear distributed BFLF2 into these cytoplasmic vesicles in co-transfected cells.

Because parts of BFRF1-induced vesicles were located in the perinuclear compartment, we wondered whether these vesicles associate with particular cellular organelles. Therefore HeLa cells were cotransfected with plasmids expressing BFRF1 and YFP-tagged organelle retention sequences, including for the ER, Golgi, and endosomes. Compared with the specific localization of the various organelles in vector transfected cells, expression of HA-BFRF1 redistributed organelle markers into an uneven and slightly clumped pattern (Figure S2A and S2B). Without a conventional organelle targeting sequence, BFRF1 was predominantly colocalized with ER markers at the nuclear rim and nucleus-extended structure. A small portion of BFRF1 was colocalized with the Golgi markers in the cytoplasm and with endosome markers at the nuclear periphery (Figure S2B, merge), suggesting that BFRF1-induced vesicles may fuse progressively with other cytoplasmic organelles.

**Figure 3:**
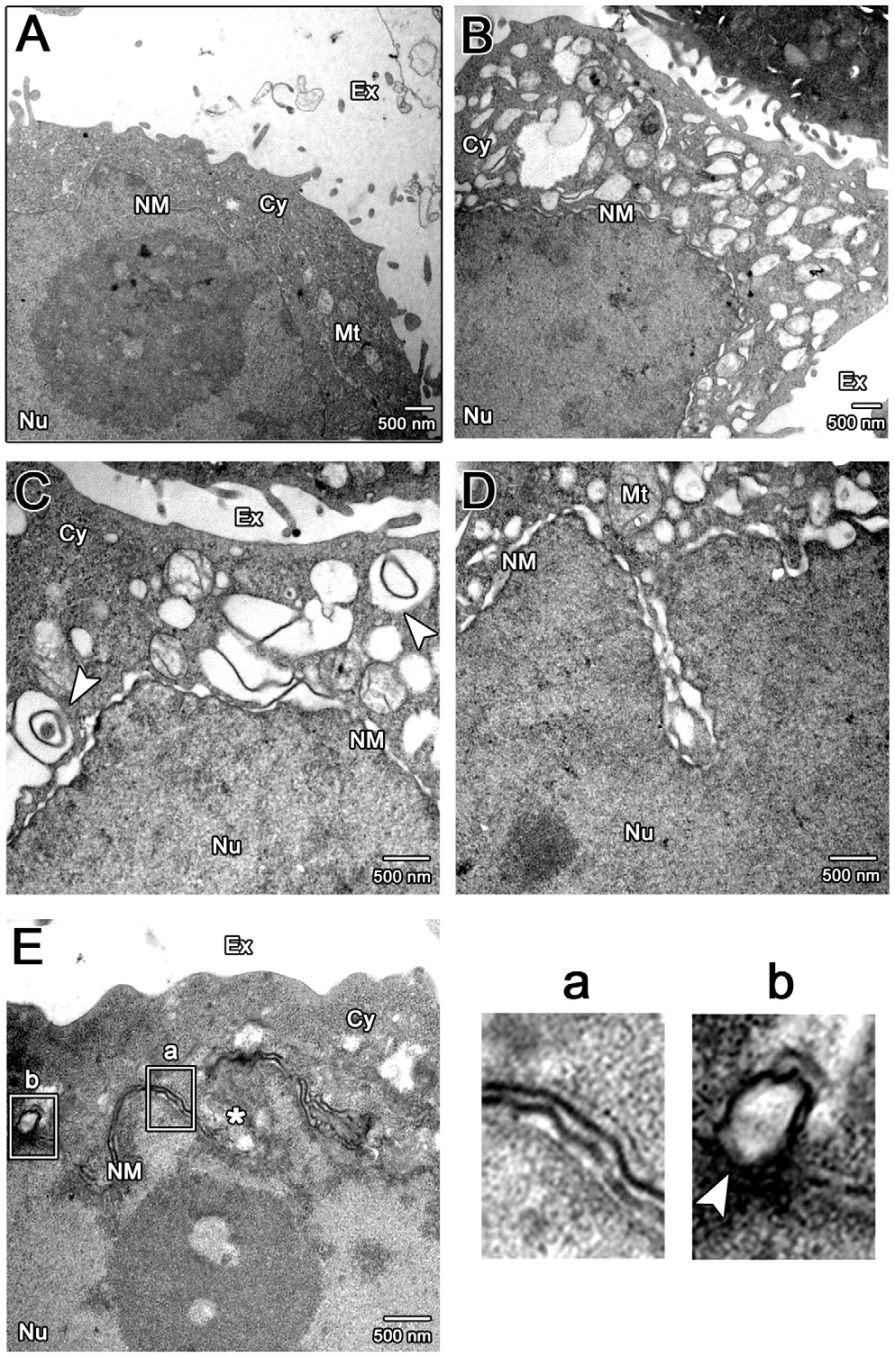
TEM analysis of cells with EBV BFRF1 expression. HeLa cells were transfected with control vector (A) or plasmid expressing HA-BFRF1 (B to E). At 24 h post transfection, cells were fixed and processed for TEM as described in Materials and Methods. More than 30 cells were observed in each set and representative nuclear and cytoplasmic regions of transfected cells are shown. Images were taken at a final magnification of ×12,000 (A and B) or ×20,000 (C to E). (**A and B**) Expression of HA-BFRF1 induced multiple irregular shape vesicles in cytoplasm (Cy) and altered nuclear membrane structure (NM). (**C**) Irregular shape vesicles with single or multiple-layer membranes (arrowhead) were observed in cells transfected with HA-BFRF1. (**D**) A wavy NM was observed in a nuclear cave structure of HA-BFRF1 transfected cell. (**E**) An untypical multilayered NM (a) and a budding nuclear membrane (b) were shown at higher magnification in the inset, respectively. “*”, indistinct margin of NM. Cy, cytoplasm; Nu, nucleus; Mt, mitochondria; Ex, extracellular space.

To determine directly the effect of BFRF1 expression on nuclear membrane alteration and vesicle formation, we visualized the subcellular structures in HeLa cells expressing BFRF1 by transmission electron microscopy (TEM). Compared to the smooth nuclear membrane and homogenous cytoplasm in vector transfected cells (Figure 3A), multiple irregular cytoplasmic vesicles and altered nuclear membranes were observed in more than 30 HA-BFRF1 expressing cells (Figure 3B). Both single- and multiple-layer membranes (arrowheads) were found in these irregular cytoplasmic vesicles (Figure 3C). The nuclear membrane showed an irregular wavy structure (Figure 3D), with cisternal multilayered membranes (Figure 3E, a) or the disappearance of margin integrity (Figure 3E, star). Notably, a budding structure, similar to retroviral budding through plasma membranes, was also observed on the modified nuclear membrane of HA-BFRF1 transfected cells (Figure 3E, b), indicating that expression of EBV BFRF1 alone is sufficient to induce vesicle formation through dramatic modulation of the nuclear envelope.

### ESCRT proteins associate with EBV BFRF1 and regulate BFRF1-induced vesicle formation from the nuclear membrane

**Figure 4:**
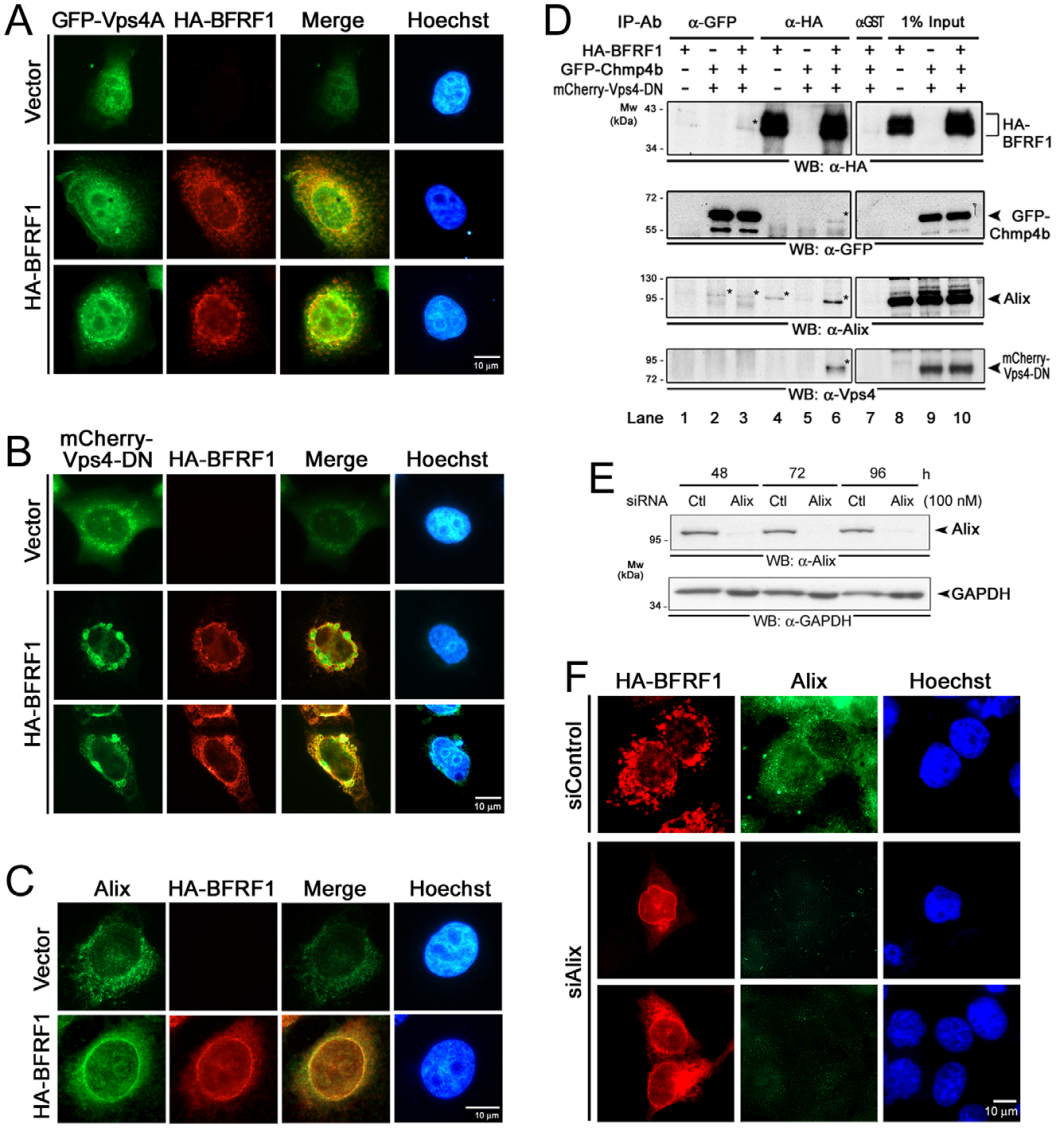
Cellular ESCRT components contribute to BFRF1-mediated vesicle formation. (**A**) Slide-cultured HeLa cells were transfected with plasmids expressing wild-type GFP-Vps4A and HA-BFRF1 for 24 h, and stained for HA (red) and DNA. (**B**) HeLa cells were transfected with plasmids expressing Vps4-DN and HA-BFRF1 for 24 h and stained for HA (red) and DNA. The original color of HA (green) and mCherry (red) were converted to red and green to facilitate interpretation of the data. Nuclear membrane associated HA-BFRF1 and Vps4-DN colocalized at nuclear-associated clusters without cytoplasmic vesicle structure (yellow, Merge). (**C**) Slide-cultured HeLa cells were transfected with plasmid expressing HA-BFRF1. At 24 h post transfection, cells were fixed and immuno-stained for HA (red) and endogenous Alix (green). Expression of BFRF1 redistributed Alix to nuclear rim. (**D**) Lysates from HeLa cells transfected with vector or plasmid expressing HA-BFRF1, GFP-Chmp4b or mCherry-Vps4-DN were immunoprecipitated with antibody against GFP, HA or GST control antibody. The immunocomplexes were then resolved by 10% SDS-PAGE and immunoblotted with antibodies against HA, GFP, Alix or Vps4. BFRF1 associated with multiple ESCRT proteins. (**E**) To check the knockdown efficiency of siRNA treatment, HeLa cells were transfected with Alix or control (Ctl) siRNA for 48, 72 or 96 h, harvested and subjected to immunoblotting analysis using anti-Alix or anti-GAPDH antibody. (**F**) HeLa cells were transfected with Alix (siAlix) or control (siControl) siRNA for 48 h and cotransfected with siRNAs and plasmid expressing HA-BFRF1 for additional 24 h. The transfected cells were then fixed and immuno-stained for HA (red) and Alix (green). Knockdown of Alix expression abolished BFRF1 induced vesicle formation.

Because the ESCRT components Chmp4b and Alix showed partial colocalization with BFRF1 at the nuclear periphery or rim of cells replicating EBV (Figure 1), we explored further the contribution of the cellular ESCRT machinery to BFRF1-mediated vesicle formation. We found wild-type GFP-Vps4A displayed diffuse cytoplasmic and nuclear fluorescence in vector transfected HeLa cells, whereas GFP-Vps4-DN accumulated as aggregates in the perinuclear space, as the characteristic class E compartment [12] (Figure 4A and 4B, upper panels). Expression of HA-BFRF1 or CFP-BFRF1 caused the redistribution of GFP-Vps4A into cytoplasmic puncta and enhanced nuclear membrane associated structures with a partial colocalization pattern (Figure 4A and S2C). Remarkably, expression of Vps4-DN abolished the BFRF1-induced vesicle formation in the cytoplasm (Figure 4B and S2D). Vps4-DN colocalized with HA-BFRF1 on the nuclear membrane as connected froth-like structures or at the nuclear periphery as clumps, suggesting that functional ESCRT machinery is required for BFRF1-induced vesicle formation.

Next, we explored the participation of ESCRT components in BFRF1 mediated vesicle formation. The distribution of the ESCRT-I protein TSG101 was first observed in HeLa cells co-expressing HA-BFRF1 and Flag-BFLF2. Using two different antibodies (r654 prefers the native epitopes and 4A10 detects a.a. 167–374 of TSG101), we found that TSG101 showed a homogenous cytoplasmic distribution in vector transfected cells and was slightly enhanced to the nuclear rim with HA-BFRF1 and Flag-BFLF2 in co-expressing cells (Figure S2E). A coimmunoprecipitation assay also supported the interactions between TSG101 and HA-BFRF1/Flag-BFLF2 complexes (data not shown), implying that TSG101 may participate in BFRF1/BFLF2-mediated functions during virus maturation.

In contrast to TSG101, immunofluorescence staining showed the ESCRT adaptor Alix was expressed predominantly in a diffuse cytoplasmic pattern in vector transfected cells (Figure 4C). With the expression of HA-BFRF1, Alix was redistributed partially and colocalized with HA-BFRF1on the nuclear rim, similar to the patterns observed in virus replicating cells (Figure 1D). Because Alix was found to bridge viral proteins to ESCRT in many cases, we suspected Alix may serve as the factor bridging BFRF1 and the cellular ESCRT machinery. We found that only small amounts of HA-BFRF1 and Alix were coimmunoprecipitated with GFP-Chmp4b by GFP antibody, even in the presence of Vps4-DN (Figure 4D, lane 2 and 3). Also, small amounts of Alix were coimmunoprecipitated with HA-BFRF1 by anti-HA antibody (Figure 4D, lane 4). Nevertheless, coexpression of Vps4-DN and GFP-Chmp4b, which presumably accumulate as unresolved class E complexes, significantly enhanced the interaction between BFRF1 and Alix (Figure 4D, lane 6), suggesting the ESCRT machinery participates in BFRF1 induced vesicle formation.

To prove the role of Alix in bridging BFRF1 and the ESCRT machinery, a highly specific Alix siRNA [40] was then added to transiently transfected cells. Compared with control siRNA, double treatments with specific siRNA significantly reduced the expression of Alix at 48, 72 and 96 h post transfection (Figure 4E). In Alix knockdown cells, BFRF1 lost the ability to induce vesicle formation and showed a nuclear rim or cytoplasmic reticular pattern (Figure 4). Nevertheless, vesicles were still observed in cells with residual Alix signals, suggesting that a small amount of Alix is sufficient for the function of the ESCRT machinery (data not shown). Interestingly, we noticed that the cytoplasmic accumulation of BFRF1 in Alix knocked down cells is different from the nuclear rim retention of BFRF1 in cells expressing Vps4-DN or GFP-Chmp4, suggesting Alix may contribute to the nuclear targeting of BFRF1.

### The ability of EBV BFRF1 to induce vesicle formation was not found for HSV-1 UL34

To determine whether the ability of BFRF1 to induce vesicle formation is conserved among other herpesviral homologs, HSV-1 HA-UL34 and Flag-UL31 were expressed in HeLa cells. As described previously [24], HA-UL34 expression alone showed a perinuclear and cytoplasmic reticular pattern without vesicle formation in the cytoplasm, whereas Flag-UL31 was expressed predominantly in the nuclei of transfected cells (Figure S3A). HA-UL34 partially redistributed the nuclear emerin to the perinuclear region, whereas emerin showed a relatively typical nuclear rim distribution in cells expressing Flag-UL31. Co-expression of HSV-1 HA-UL34 and Flag-UL31 produced a colocalization pattern at the perinuclear puncta and thickened nuclear rim (Figure S3B), which is considered to result from nuclear envelope alteration induced by UL34/UL31 complexes [41]. In a sequence alignment, unique regions were found in EBV BFRF1 and HCMV UL50, in addition to the conserved domains (Figure S3). Altogether, data here suggest other BFRF1 homologs also modulate the architecture of the nuclear envelope, while the vesicle forming ability may not be conserved in BFRF1 homologs.

### Multiple domains of BFRF1 are responsible for Alix interaction, nuclear membrane modulation and vesicle formation

**Figure 5:**
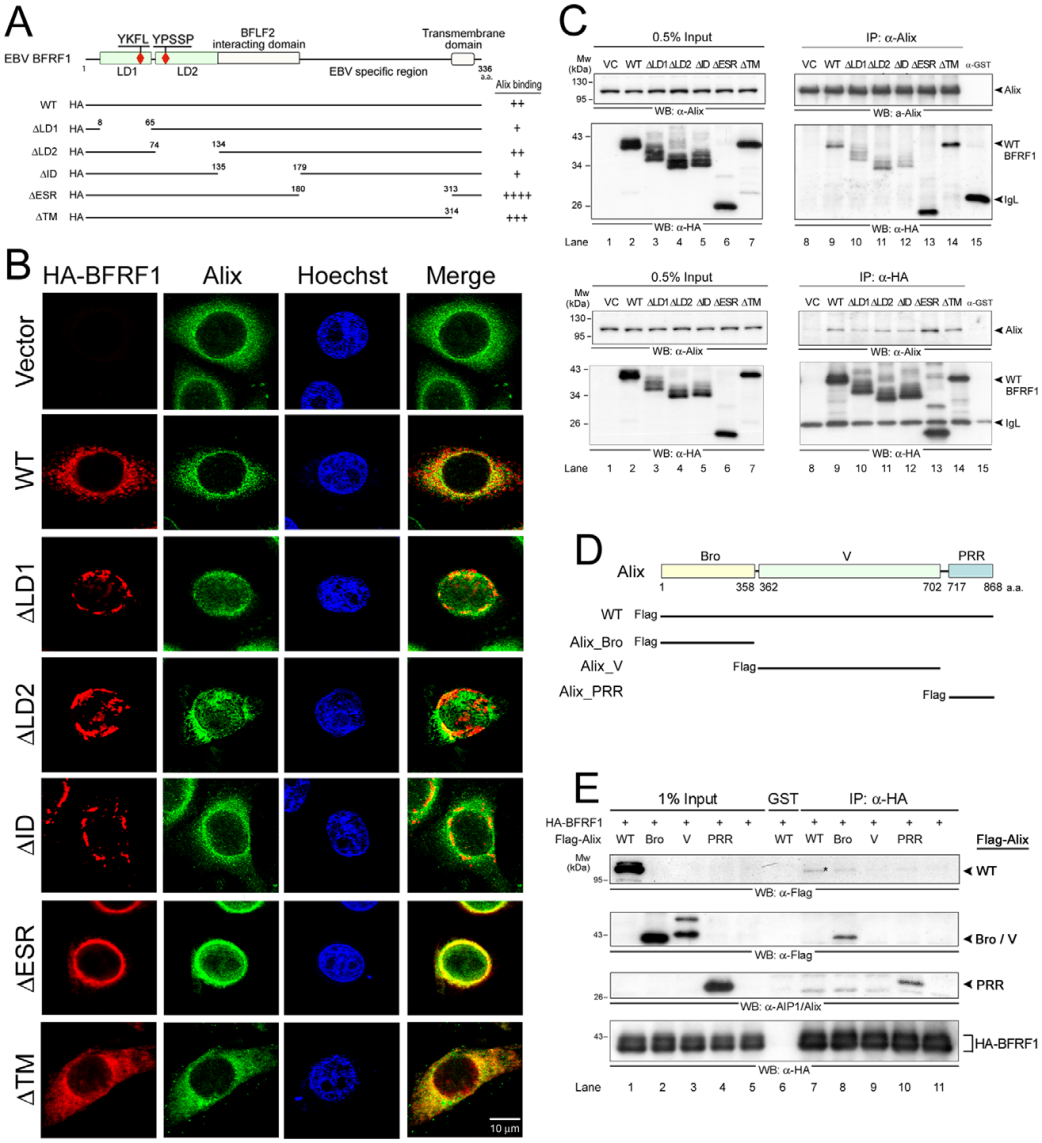
Functional domain mapping of BFRF1 and Alix required for the vesicle formation and molecular interaction. (**A**) Schematic representation of HA-tagged BFRF1 mutants. Putative late domain, BFRF2 interacting domain, EBV specific region and transmembrane domain were predicted by MUSCLE and ClustalW2 multiple alignment (http://www.ebi.ac.uk/Tools/msa/muscle/and/clustalw2/), and DAS protein structure modeling (http://www.sbc.su.se/~miklos/DAS/) programs. A series of BFRF1 deletion mutants, including ΔLD1, ΔLD2, ΔID, ΔESR and ΔTM, were generated as indicated. The relative Alix binding ability was evaluated in coimmunoprecipitated protein signals, and summarized in the right-hand column. (**B**) Slide-cultured HeLa cells were transfected with HA-BFRF1 wild-type (WT), ΔLD1, ΔLD2, ΔID, ΔESR or ΔTM-expressing plasmids, fixed by 4% paraformaldehyde, stained for HA (red), Alix (green) and DNA and observed in confocal microscopy. (**C**) Lysates from HeLa cells transfected with HA-pSG5 vector (VC) or plasmid expressing WT, ΔLD1, ΔLD2, ΔID, ΔESR or ΔTM of HA-BFRF1 were immunoprecipitated with antibody against Alix (upper panel), HA (lower panel) or anti-GST control antibody. The immunocomplexes were then resolved by 10% SDS-PAGE and immunoblotted with antibodies against Alix and HA. IgL, immunoglobulin light chain. (**D**) Schematic representation of Flag-tagged Alix mutants. Alix functional domain mutants, including Alix_Bro, Alix_V and Alix_PRR were used as indicated. WT, wild type; Bro, Bro1 domain; V, “V” domain; PRR, proline-rich region. (**E**) WT, Bro and PRR fragments of Alix were coimmunoprecipitated with HA-BFRF1. Lysates from HeLa cells transfected with vector or plasmid expressing HA-BFRF1 coupled with Flag-Alix, Flag-Alix_Bro, Flag-Alix_V or Flag-Alix_PRR. HA-BFRF1 were immunoprecipitated with antibody against HA or GST. The immunocomplexes were then resolved by 10% SDS-PAGE and immunoblotted with antibodies against Flag, Alix, or HA.

Here, BFRF1 appears to be the first nuclear envelope associated protein using the ESCRT machinery through interaction with Alix. To characterize the region responsible for interaction with Alix, vesicle formation and membrane anchoring, we analyzed the BFRF1 protein sequence through motif search and alignment with other herpesviral homologs. No conventional L motifs (PTAP, PPXY and YXXL) were identified in BFRF1, whereas two putative L-like motifs (_62_YKFL_65_ and _74_YPSSP_78_) were found within the amino terminal putative late domain 1 (LD1, a.a. 8–65) and LD2 (a.a. 74–134) of BFRF1. In addition, a putative BFLF2 interacting domain (ID, a.a. 135–179) and a transmembrane region (TM, a.a. 314–336) were identified in the BFRF1 protein by multiple alignments with herpesviral homologs and protein secondary structure prediction (Figure 5A and S3C). An EBV specific region (ESR) also was identified in the region a.a. 180–313 of BFRF1. A series of HA-tagged BFRF1 deletion mutants was subsequently generated to test the functions of various regions.

In confocal images, Alix located predominantly in the cytoplasm of vector transfected HeLa cells. Expression of BFRF1 slightly redistributed Alix close to the nuclear rim and BFRF1 was partially associated with Alix in nuclear peripheral vesicles (Figure 5B, merge). The BFRF1ΔLD1, ΔLD2 and ΔID proteins showed fragmented, aggregated patterns around the nucleus or were embedded in the nuclear membrane. Partially Alix-colocalized signals were observed in the nuclei of cells expressing BFRF1ΔLD2 and at the nuclear rim of cells expressing BFRF1ΔLD1 or ΔID. In contrast, BFRF1ΔESR was colocalized with Alix at the nuclear rim in a continuous smooth pattern, whereas BFRF1ΔTM is evenly distributed with Alix in the cytoplasm. It was notable that BFRF1-induced vesicles were abolished in all mutants (Figure 5B), suggesting multiple regions of BFRF1 are required for vesicle formation. The EBV specific region of BFRF1 (a.a. 160–313) may mediate the membrane budding and the putative TM is responsible for its nuclear envelope targeting. In confirming the BFRF1-Alix interaction domain, slow migrating bands in addition to the predicted molecular weight, which may resemble post translational modification, were seen clearly for ΔLD1, ΔLD2 and ΔID of BFRF1. Compared with the WT, reduced amounts of BFRF1ΔLD1, ΔLD2 or ΔID were coimmunoprecipitated with Alix in both directions (Figure 5C, lanes 9 to 12), suggesting the region between LD1 and ID may mediate the interaction with Alix. Interestingly, deletion of ESR or TM enhanced the interaction between BFRF1 and Alix (Figure 5C, lane 13 and 14), suggesting without the sequential recruitment of the ESCRT proteins for vesicle formation, the abrogation of the membrane modulating or anchoring function of BFRF1 is likely to stabilize the interaction between BFRF1 and Alix.

The ability of BFRF1 mutants to reorganize the nuclear membrane was also investigated using the INM-anchoring emerin as a marker in confocal analysis (Figure S4A). Expression of WT BFRF1 induced the redistribution of emerin into small cytoplasmic puncta with partial colocalization with BFRF1. Expression of BFRF1ΔLD1, ΔID and ΔESR partially redistributed emerin to perinuclear clumps or into the nucleus with dispersed staining, whereas BFRF1ΔLD2 induced the BFRF1-emerin clumping in the nucleus. Interestingly, BFRF1ΔTM caused partial redistribution of emerin into the cytoplasm, suggesting BFRF1ΔTM can still disturb the integrity of the nuclear membrane. It is possible that BFRF1 modified the membrane architecture through direct or indirect interactions with other membrane-anchoring proteins.

Next, the BFRF1-interacting regions in Alix were revealed. So far, three domains were identified in Alix, the amino terminal Bro domain (a.a. 1–358), the central V domain (a.a. 362–705) and the carboxyl-terminal Proline-rich region (PRR, a.a. 717–868) (Figure 5D) [42]. Using Flag-tagged functional fragments of Alix, we found that, with a small amount of the WT, the Bro and PRR domains, but not V, were coimmunoprecipitated with HA-BFRF1 (Figure 5E, lanes 7 to 10), suggesting there could be more than one contact region between BFRF1 and Alix. Again, the Bro and PRR domains alone appeared to form a more stable complex with BFRF1, compared to that of WT Alix.

In the current model of membrane morphogenesis, the intermolecular interactions of membrane anchoring proteins provide the force to curve the cellular membrane [43], [44]. To determine the potential of BFRF1 to induce membrane curving, we detected the possible intramolecular interaction of BFRF1 by immunoprecipitation. In reciprocal immunoprecipitation of HA-BFRF1 and GFP-BFRF1, we showed that BFRF1 can form dimers (Figure S4B, lane 8), potentially to force the membrane curving.

### Alix siRNA, dominant negative Vps4 and BFRF1 mutants cause the accumulation of viral DNA and major capsid proteins in the nucleus

**Figure 6:**
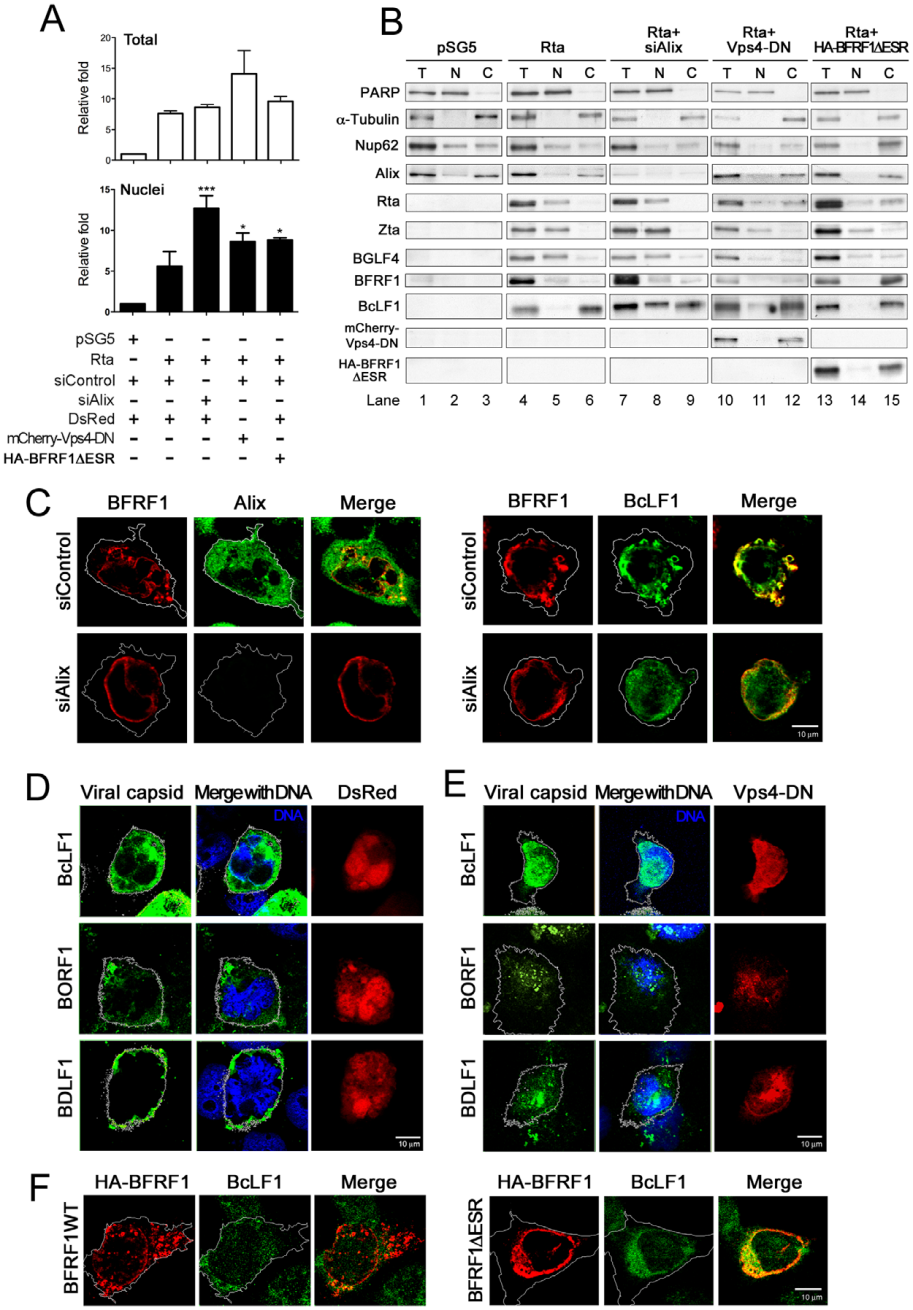
Expression of Alix siRNA, dominant negative Vps4 or BFRF1ΔESR accumulates viral genome and capsid proteins in the nucleus. (**A and B**) To observe the effect of Alix siRNA, BFRF1ΔESR or Vps-DN expression on the viral genome and distribution of capsid proteins, NA cells were transfected with plasmid expressing Rta or vector pSG5 and siAlix or siControl for 48 h and followed by another transfection of siRNA or plasmid expressing mCherry-Vps4-DN, BFRF1ΔESR, or related control vector, and incubated for additional 48 h. The subcellular fractionation was then performed as described in Materials and Methods. DNA and protein were extracted individually from transfected cells into total (T, or intracellular), nuclear (N) and cytosol (C) fractions. The extracted DNA in the total and nuclear fraction was then subjected to qPCR analysis targeting the EBV DNA *Bam*HI W fragment and the relative folds of viral genome in intracellular or nuclear compartment of transfected cells are indicated in (A). Results are means ± standard deviations from two separate transfections. Data are representatives of two independent experiments. The statistically significant differences between Rta expression alone and coexpression of Rta and siAlix, mCherry-Vps4-DN or HA-BFRF1ΔESR were calculated by the paired Student's t-test and are indicated at the top of the bars. *, P<0.05; ***, P<0.001. The protein expression in the various factions is shown in (B). PARP and α-Tubulin serves as nuclear and cytosolic markers, respectively. (**C**) NA cells were transfected with plasmid expressing Rta together with siAlix or siControl for 48 h and followed by another transfection of siRNA for additional 24 h. The cells were fixed by 4% paraformaldehyde and immune-stained for Alix, BFRF1 and capsid protein BcLF1, respectively. The margin of the cells was detected by MetaMorph software and is indicated by a white outline. Treatment with siAlix induced the accumulation of BcLF1 in the nucleus. (**D and E**) To observe the localization of nucleocapsids, lytic NA cells with DsRed control (red) or mCherry-Vps4-DN (red) expression were fixed at 72 h post transfection, immuno-stained for the subcellular distribution of major viral capsid components BcLF1, BORF1 or BDLF1 (green) with anti-BcLF1 L2, anti-BORF1 or anti-BDLF1 antibody, and observed by confocal microscopy. Cellular DNA was detected by Hoechst 33258 and merged with capsid protein staining. Expression of Vps4-DN promoted the intra-nuclear accumulation of capsid components BcLF1, BORF1 and BDLF1 (overlaid with blue DNA signals). (**F**) NA cells were transfected with plasmid expressing Rta for 48 h followed by transfection of HA-BFRF1 or BFRF1ΔESR plasmid for an additional 24 h. Transfected cells were fixed, immuno-stained for viral major capsid component BcLF1 (green) and HA (red) and observed by confocal microscopy. BFRF1ΔESR of expression reduced the BcLF1 detection in cytoplasm of transfected cells.

We determined next whether the recruitment of ESCRT machinery by BFRF1 contributes to the nuclear egress of EBV nucleocapsids. In our subcellular fractionation analysis, PARP and α-Tubulin were clearly separated into the nuclear and cytosolic fractions (Figure 6B, lanes 1 to 3). Nucleoporin Nup62 was separated into both nuclear and cytoplasmic fraction as described [45], indicating the protocol is suitable for identifying the components of different subcellular compartments. Simultaneously, the amounts of viral genome were first detected by quantitative PCR in reactivated NA cells in the presence of Alix siRNA or a plasmid expressing Vps4-DN (Figure 6A). At 72 h post transfection, EBV reactivation enhanced the intracellular viral genome content by 7.5 fold (total) and 5 fold in the nuclear fraction. Treatment with Alix siRNA did not change the amount of intracellular viral genomes significantly but this was enhanced slightly by the expression of Vps4-DN, similar to the observation in Figure 1A. Remarkably, treatment with siAlix increased intranuclear viral genome content of Rta transfected NA cells by more than 2 fold. Expression of Vps4-DN also increased the intranuclear viral genome content (∼1.3 fold), suggesting that Alix and functional ESCRT machinery contribute to the nucleus to cytosol translocation of the EBV genome. As well as the viral genome, we also analyzed protein distribution in these cells by subcellular fractionation (Figure 6B). Compared to Rta plasmid transfection alone (Figure 6B, lanes 4 to 6), additional treatment with siAlix led to significant accumulation of the major capsid protein BcLF1 in the nuclear fraction (Figure 6B, lane 8). With a 50∼60% transfection percentage, expression of Vps4-DN also increased the detection of BcLF1 in the nuclear fraction (Figure 6B, lanes 13 and 15), suggesting that functional ESCRT machinery regulates the nucleus to cytosol translocation of the major capsid protein.

Because the replication efficiency of EBV is much lower than that of alpha- or beta-herpesviruses, we thought it would be difficult to quantify the intranuclear viral capsids using EM. Therefore, antibodies against viral capsid proteins were used to locate the viral capsids in cells replicating EBV. To this end, EBV-positive NA cells were cotransfected with Alix siRNA or plasmids expressing Vps4-DN and Rta and stained for Alix, BFRF1, viral capsid component(s) and cellular DNA. In the immunofluorescence images, BFRF1 located predominantly at the nuclear rim surrounding the cellular DNA, with punctate structures in the cytoplasm of NA cells at 72 h post Rta transfection (Figure S5A, vector). In the presence of siRNA control, the major capsid protein BcLF1 colocalized with BFRF1 in the cytoplasmic punctate structures (Figure 6C, right panels). Knockdown of Alix caused the accumulation of BcLF1 in the nucleus. In addition, expression of Vps4-DN restricted BFRF1 distribution to the nuclear membrane and enlarged perinuclear structures (Figure S5, mCherry-Vps4-DN) which may be derived from the extended nuclear envelope, such as the intranuclear nuclear reticulum or perinuclear ER compartment [46]. The viral capsid proteins, including BcLF1 and triplex proteins BORF1 and BDLF1, also were detected to indicate the likely distributions of viral nucleocapsids. In cells expressing control DsRed protein, we found that viral capsid proteins were detected predominantly in the cytoplasm or at the margin of Rta-transfected NA cells at 72 h post transfection (Figure 6D), suggesting the nucleocapsids may translocate through the cytoplasm for virion release. Remarkably, expression of Vps4-DN significantly promoted the accumulation of the viral capsid components BcLF1, BORF1 and BDLF1 in the nucleus (Figure 6E, overlaid with light green signals), suggesting that the ESCRT machinery regulates EBV maturation, very likely at the nuclear egress of nucleocapsids.

Finally, because the ESR region is unique in EBV BFRF1 and BFRF1ΔESR shows strong colocalization with Alix at the nuclear rim, without vesicle formation (Figure 5B), we sought to determine whether expression of the BFRF1ΔESR mutant interferes with virus maturation. We found that coexpression of BFRF1ΔESR with Rta in NA cells did not affect the amount of intracellular EBV genomes (Figure 6A, last columns). Notably, BFRF1ΔESR expression increased the content of intranuclear viral genomes. In protein analysis, BFRF1ΔESR was co-fractionated with Nup62, EBV Rta, BFRF1 and BcLF1 in the transfected cells (Figure 6B, lanes 10 to 12), implying that BFRF1ΔESR may trap viral DNA, nuclear envelope components and viral capsids in the cytoplasm. Consistently, BFRF1ΔESR not only induced the accumulations of Alix and emerin but also colocalized with capsid BcLF1 protein in a distinct perinuclear compartment, without cytoplasmic vesicle-like structures (Figure S5B and S5C). In contrast, expression of WT BFRF1 seemed to enhance vesicle formation and the cytoplasmic BcLF1 staining pattern in reactivated NA cells (Figure 6F and S5C). Overall, we assumed the coordination of multiple domains within BFRF1 is critical for recruiting the ESCRT machinery during viral nuclear egress; therefore BFRF1ΔESR may function as a dominant negative mutant for EBV maturation.

## Discussion

In this study, we aimed to explore the contribution of the ESCRT machinery to the maturation process of EBV. We provide evidence that EBV BFRF1 is the first identified nuclear envelope-associated protein that employs the cellular ESCRT machinery to induce nuclear envelope-derived cytoplasmic vesicles. Evidence here also suggests that a similar mechanism is used for the nuclear egress of EBV nucleocapsids.

Using dominant negative mutants, we showed that the ESCRT machinery is important for the maturation of EBV virions (Figure 1A). In cells replicating the virus, the ESCRT proteins were redistributed and partially colocalized with the viral BFRF1 protein at nuclear rim. Interestingly, the INM-associated proteins, lamin A/C and emerin, and some cellular DNA, were detected simultaneously within cytoplasmic BFRF1 associated punctate structures (Figure 1F and 1G). In the subsequent immuno-staining and transmission EM analysis, we found that expression of BFRF1 alone is sufficient to modulate nuclear membrane structure and induce vesicle formation (Figure 2 and 3). However, the dispersed distribution of BFRF1-induced vesicles in HeLa cells was different from the polar distribution of BFRF1-containg puncta near the nuclear concave of EBV reactivated cells, suggesting that other viral proteins also may regulate the BFRF1-contaning structures during virus replication. Remarkably, BFRF1 alone was sufficient to recruit and interact with the ESCRT proteins Alix, Chmp4b and Vps4 (Figure 4). Expression of Alix siRNA or the dominant negative form Vps4 abolished BFRF1-induced vesicle formation from the nucleus-associated membrane. Because of the low replication efficiency of EBV in the epithelial cells, we were not able to quantify nucleocapsids directly under EM. However, Alix siRNA, Vps4-DN and the BFRF1ΔESR mutant accumulated viral DNA and major capsid components in the nuclei of cells replicating the virus (Figure 6).

**Figure 7:**
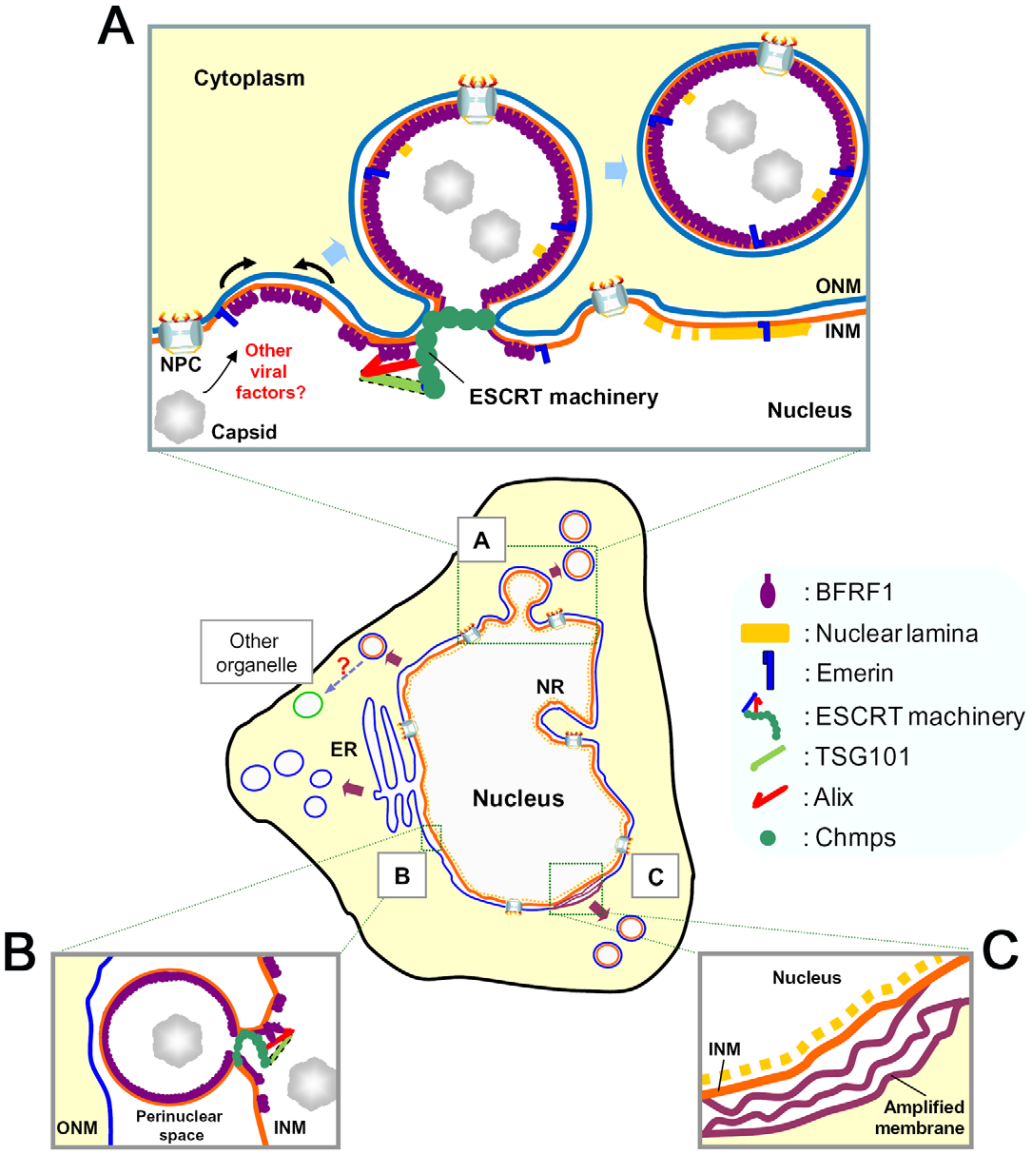
A hypothetical model of the interaction among EBV BFRF1 and cellular ESCRT components in vesicle formation and modulation of the nuclear membrane. After EBV reactivation, membrane anchoring BFRF1 targets to the nucleus associated membranes. BFRF1 potentially regulates the membrane through protein oligomerization and then recruits Alix and, sequentially, other ESCRT components to derive the vesicles from the double layer nuclear envelope (**A**), outer nuclear membrane (ONM) or from inner nuclear membrane (INM) (**B**). These nuclear membrane-derived vesicles may contain some nucleoporins and other INM proteins, such as lamin A/C or emerin. Cooperating with other viral factors, such as BFLF2, the BFRF1-mediated vesicles may further recruit and pack some nucleocapsids for the subsequent nuclear egress. Additionally, the expression of BFRF1 induces the formation of multilayered cisternal nuclear membranes (**C**), which may provide more membranous structure for viral budding. ER, endoplasmic reticulum. NPC, nuclear pore complex.

A hypothetical model of the coordinated action of EBV BFRF1 and cellular ESCRT components in modulating the nuclear membrane is proposed (Figure 7) according to our observations and the electron tomography (ET) images from MHV-68 (Figure 6 in [35]). We postulate that, after EBV reactivation, the membrane-anchoring BFRF1 may be translocated from the ER membrane to the nuclear membrane and subsequently initiate the protrusion of the nucleus associated membranes. BFRF1 then cooperates with the ESCRT proteins to form vesicles, which may be derived from double- (Figure 3C and 7A) and single-layered nuclear membrane (Figure 3B, 3C and 7B). In one way, the vesicles budding from the INM may be subsequently fused with the ONM as the scenario proposed for HSV-1 [13], [14]. The vesicles derived from the double-layered nuclear envelope or INM may contain some cellular nuclear components, such as lamin, emerin and even cellular DNA fragments. BFRF1 expression also induces the amplification of nuclear membranes, which potentially may provide more membranous materials for viral maturation (Figures 3E and 7C). In cells replicating EBV, other viral factors are believed to cooperate with BFRF1, regulate the function or subcellular localization of ESCRT components and aid packaging of nucleocapsids into nuclear membrane derived vesicles for nuclear egress.

In our model, the mechanism through which BFRF1 and Alix translocate into the nucleus remains unclear. Recently, multiple pathways, such as nuclear pore complex (NPC)/Ran-GTP dependent pathway, a diffusion-retention mechanism and nuclear envelope trafficking have been shown to mediate the trafficking of various cellular integral and peripheral INM proteins [47]. Based on EM observations of the nuclear egress of HSV-1, a putative NPC-independent vesicular trafficking pathway was proposed for the newly synthesized ONM-associated viral proteins to travel through the nuclear envelope lumen and subsequently fuse to INM [47], [48]. In our study, we found Alix was distributed in the cytoplasm in cells not replicating EBV, while EBV reactivation or expression of BFRF1ΔESR redistributed a portion of Alix into the nucleus (Figure 1D and 5B). Alix siRNA caused the accumulation of BFRF1 on the nuclear membrane or ER-associated apparatus in a reticular pattern in the cytoplasm, implying that Alix and BFRF1 may regulate their nuclear transport reciprocally (Figure 6C). One possibility is that newly synthesized BFRF1 can oligomerize on the ONM and form a structure protruding towards the nuclear envelope lumen, then small vesicles may be formed through the recruitment of ESCRT components into the nuclear envelope lumen, and subsequently fuse with the inner nuclear membrane. Once BFRF1 is translocated onto the INM, the recruited Alix would then be released into the nucleus. There could be a reinitiated assembly of BFRF1, Alix and other ESCRT components to promote the large vesicle formation towards the cytoplasm. This scenario is supported partly by the observation of large double membrane invaginations, containing multiple nucleocapsids and nuclear pore complexes, in the ET images of cells replicating MHV-68 (Figure 6 in [35]). Further studies are needed to solve the various questions, including how virus controls the budding from single- or double-layered nuclear membranes, how the transport direction is controlled in cells replicating the virus, and whether the same ESCRT components are involved in both directions of vesicle formation. In addition, according to our organelle maker images, the BFRF1-induced nuclear envelope-derived vesicles may fuse with the cytoplasmic apparatus to change the membrane composition and promote tegumentation and, finally, viral maturation. Techniques such as immunogold-labeling, together with high-resolution imaging, may determine whether BFRF1containg cytoplasmic vesicles can use the ESCRT machinery to modulate cytoplasmic organelles.

Regarding the functional domains, we found that the Bro- and PRR- regions of Alix showed stronger interactions with BFRF1 than that of the wild type Alix (Figure 5E). Because the wild type Alix is involved in a very transient mode in coordinated association/dissociation among different domains for its function [49], we suspect the interaction of a single domain of Alix with BFRF1 tends to be stabilized. In the functional domain mapping, LD1 (a.a. 8–65), LD2 (a.a. 74–134) and ID (a.a. 135–179) of BFRF1 are required for vesicle formation (Figure 5B). Alix and emerin showed distinct subcellular distribution in the presence of BFRF1ΔLD1, ΔLD2 or ΔID (Figure 5B and S4A), suggesting that these regions are required for functional recruitment of ESCRT proteins and nuclear membrane remodeling. Further characterization of the functional motifs LD1, LD2 and ID (a.a. 8–179) of BFRF1 may suggest possible mechanisms involved in the vesicle formation process. However, unlike ΔLD1, ΔLD2 and ΔID, ΔESR was strongly co-stained with Alix on the nuclear rim and induced intranuclear distribution of emerin (Figure 5B and S4A). Collectively, these data indicate that different domains of BFRF1 may function at different stages of the vesicle formation process. In cells with BFRF1 homolog expression or herpesviruses replication, multilayered nuclear membranes, packaged cisternal and annulate lamellae structures are observed commonly (Figure 7C) and [18], [22], [50]. We speculate that uncoordinated interactions between the ESCRT proteins and membrane anchoring BFRF1 homologs may induce the formation of multilayered class E compartment-like structures. Further EM studies may be required to reveal how BFRF1 derived vesicles traffic in the cytoplasm to facilitate the cytoplasmic maturation of EBV.

Interestingly, the ability to induce vesicle formation was not found in the HSV-1 BFRF1 homolog, pUL34, in this study. Other studies also showed that the coexpression of pUL34 and pUL31 of pseudorebies virus or ORF67 and ORF69 of Kaposi's sarcoma-associated herpesvirus (KSHV) in transfection system is required for the formation of vesicles at the nuclear margins, resembling the primary envelopment without nucleocapsids [41], [51]. According to our sequence alignment, the functional domains LD1, LD2 and ID for ESCRT protein recruitment, and the TM domain for INM anchoring of BFRF1, are relatively well conserved among the other BFRF1 homologs (Figure S3C), suggesting that other BFRF1 homologs may share the abilities of recruiting ESCRT components and targeting to INM to facilitate nucleocapsid egress. Although HCMV pUL50 is more closely related to HSV-1 pUL34 in the phylogenic analysis (Figure S3D), a region of similar length in HCMV pUL50 and that matched the ESR of BFRF1 was identified in the alignment. Whether pUL50 also shares a similar function with BFRF1 remains to be studied.

There are some differences in the process of membrane budding of RNA viruses and the nuclear egress of herpesviruses. Most RNA viruses bud from the single lipid-bilayer cytoplasmic membrane [12], [52], [53]. In contrast, the nuclear egress of herpesviruses involves passing through double lipid-bilayer membranes and the underlying nuclear lamina network. In this study, we provide evidence that EBV redistributes several ESCRT proteins to perinuclear puncta or clumping structures (Figure 1C and S1B) to modify the nuclear membrane. Inhibition of recruited ESCRT machinery caused the obvious accumulation of viral DNA and capsid proteins in the nucleus of cells replicating EBV (Figure 6), suggesting that the cellular ESCRT machinery is required for the nuclear egress of EBV. The ESCRT machinery was shown to regulate the secondary envelopment of HSV-1 and HCMV virions in the cytoplasmic compartment [7]–[9], [16], [54]. It will be interesting to determine whether the ESCRT machinery is also involved in the nuclear egress of alpha- and beta-herpesviruses.

In terms of component usage, different herpesviruses show various dependencies on ESCRT components for their maturation. The production of HSV-1 requires multiple Chmps proteins and Vps4, but not TSG101 and Alix proteins [7], [8]. Similarly, Chmp1A and Vps4, but not TSG101 and Alix, are important for HCMV production [9]. In EBV, expression of dominant negative Chmp4b and Vps4 also reduced virion release into the culture supernatant (Figure 1A). In confocal analysis, Alix, Chmp4b, Chmp1b and Vps4 were recruited and partially colocalized with BFRF1 at the nuclear membrane, suggesting that functional Chmp proteins and Vps4 ATPase are commonly used for the maturation of herpesviruses. Different herpesviruses show varying preferences in using class E components. Here we found that BFRF1 alone was sufficient to recruit the class E proteins Alix, Chmp4 and Vps4 (Figure 4). However, when BFRF1 was co-expressed with BFLF2, only small amounts of BFRF1 were coimmunoprecipitated by TSG101 antibody (data not shown) and this interaction was not seen in BFRF1 expressing cells, suggesting that the cooperation of BFRF1 with other viral proteins may lead to highly-ordered complexes for recruiting encapsidated nucleocapsids to the budding site.

Taken together, we show that the cellular ESCRT machinery is recruited by EBV BFRF1 and participates in the scission of the nucleus-associated membrane, a mechanism used for the nuclear egress of EBV. Although the detailed coordination of ESCRT components with viral factors in cells replicating the virus needs to be studied further, this analysis provides not only information for EBV maturation, but also novel insights into the involvement of ESCRT machinery in regulating nuclear envelope architecture.

## Materials and Methods

### Cell culture, virus induction and transfection

HeLa cells were derived from human cervical epithelial cells (ATCC #CCL-2). The EBV negative cell line NPC-TW01 was established from a Taiwanese nasopharyngeal carcinoma [55] and NA is a recombinant Akata EBV converted NPC-TW01 cell line [36]. All cells were cultured in Dulbecco's Modified Eagle's Medium (HyClone) and supplemented with 10% fetal calf serum, penicillin (100 U/ml) and streptomycin (100 µg/ml) at 37°C with 5% CO_2_. For EBV induction, NA cells were transfected with plasmid pRTS15 expressing Rta [56], using Lipofectamine (Invitrogen) in OptiMEM medium (GIBCO-BRL) according to the manufacturer's instruction. For Alix siRNA treatment, Alix-specific siRNA (100 nM final) with 2′-*O*-methyl ribosyl modifications, which reduce off-target transcript silencing [40], and a sense sequence 5′-GAAGGAUGCUUUCGAUAAAUU-3′ [8] or control siRNA were synthesized by Dharmacon Research Inc. (Lafayette, CO) and transfected twice into slide-cultured cells for 48, 72 or 96 h.

### Isolation of secreted EBV particles, DNA extraction and quantitative real-time PCR (qPCR) analysis

EBV positive NA cells were transfected with vector pSG5 or Rta expressing plasmid pRTS15 accompanied by a plasmid expressing GFP-Chmp4b or Vps4-DN. At 96 h post transfection, the culture supernatants were collected, subjected to centrifugation at 10,000× g for 30 min at 4°C and filtered through a 0.45 µm nylon membrane (MCE membrane, Millipore) to remove cell debris. To harvest the secreted viral particles and extract the viral genome from the virions, the supernatant was incubated with DNase I to eliminate contamination with cellular DNA, as described previously [57]. The viral genome was concentrated using a QIAmp MinElute Virus Spin Kit (QIAGEN) according to the manufacturer's instructions. The samples were confirmed for the absence of glyceraldehyde-3-phosphate dehydrogenase DNA to rule out the possibility of DNA contamination from cell debris. The cells in the plates were lysed with DNA extraction buffer (10 mM Tris-HCl, pH 8.0, 2.5 mM MgCl_2_, 1% Tween 20, 1% NP-40, 1 mg/ml proteinase K) at 50°C for at least 4 h. To measure the viral DNA content of the transfected cells or culture supernatants, qPCR using SYBR green I dye (Invitrogen) and the iCycler iQ Detection System (Bio-Rad) was used to detect the EBV *Bam*HI W fragment and beta microglobulin (Bm) gene in the human genome (modified from [58]). Briefly, the amplification of the Bm gene was used to determine the input cellular DNA in serial dilutions (10^4^, 10^3^, 10^2^, 10^1^ and 1 cells) of standard EBV positive H2B4 cells and each sample. A *Bam*HI W calibration curve also was obtained by the amplification of serial dilutions (in water) of H2B4 DNA containing 10^4^, 10^3^, 10^2^, 10^1^ and 1 *Bam*HI W copies (assuming diploid H2B4 cells carry a single copy of the EBV genome). A linear calibration curve was then generated by plotting Ct values (y-axis) against Log_10_ *Bam*HI W copy number (X-axis), from which the number of EBV genomes in the individual samples was determined. All standards and samples, together with EBV-positive and EBV negative controls, were analyzed in duplicate. The primers for Bm are 5′-GGTTGGCCAATCTACTCCCAGG-3′ and 5′-GCTCACTCAGTGGCAAAG-3′.

### Immunoblotting of EBV proteins

Rta-transfected NA cells with were lysed with radioimmunoprecipitation assay (RIPA) buffer [50 mM Tris/HCl, pH 7.5, 150 mM NaCl, 1% Nonidet P-40, 0.5% sodium deoxycholate, 0.1% SDS, complete protease inhibitor cocktail (Roche) and 1 mM Na_3_VO_4_], disrupted in SDS-sample buffer and displayed by 10% SDS-PAGE for immunoblotting detection. To detect the EBV proteins, the lab-made anti-Zta 4F10, anti-Rta 467, anti-BMRF1 88A9, anti-BGLF4 2616, anti-BcLF1 L2, and anti-BLLF1 201 were used as described previously [59]. The anti-BFRF1 and anti-BFLF2 antibodies were kindly provided by Dr. Alberto Faggioni (Università La Sapienza, Italy). For lamin A, GFP tagged proteins, mCherry-Vps4-DN or nucleoporin Nup62 detections, anti-lamin A/C 636 (Santa Cruz), anti-GFP JL-8 (Clontech), anti-Vps4 H-165 (Santa Cruz) and FG-repeat specific mAb414 (Abcam) were used as instructed.

### Plasmids construction

HA-BFRF1 and Flag-BFLF2 were generated by cloning *Xho*I-HA-BFRF1-*Not*I or *Bam*HI-Flag-BFLF2-*Not*I into pcDNA3.0 (Clontech) and were kindly provided by Dr. Hsiu-Ming Shih (Academia Sinica, Taiwan). GFP-BFRF1, CFP-BFRF1 and YFP-BFLF2 were also cloned using the same strategy into pEGFP-C1, pECFP-C1 and pEYFP-C1 (Clontech). pCR3.1-GFP–Chmp4b, pCR3.1-GFP–Vps4A and pCR3.1-Vps4-DN–mCherry [12], [60] are gifts from Dr. Paul D. Bieniasz (Rockefeller University, US). Cellular organelle marker plasmids, including pEYFP-ER, pEYFP-Golgi and pEYFP-Endo were provided by Dr. King-Song Jeng (Academia Sinica, Taiwan). For HSV-1 UL34 and UL31 expression, HA-UL34 and 3×Flag-UL31 were generated by cloning *EcoR*V-UL34-*Xho*I or *Hind*III-UL31-*EcoR*I from the strain F HSV-1 (ATCC #VR-733) infected A549 cells DNA extract into pcDNA3.0-HA or p3×FLAG-CMV-7.1 (Sigma-Aldrich), respectively. Plasmids expressing Flag-Alix, Flag-Alix_Bro, Flag-Alix_V and Flag-Alix_PRR were generated by PCR cloning of Alix gene fragments into pCAGGS/MCS vector [10]. All HA-BFRF1 mutants including HA-BFRF1d(8–65), HA-BFRF1d(74–134), HA-BFRF1d(135–179), HA-BFRF1d(180–313) and HA-BFRF1d(314–336) were generated by a single primer based site-directed mutagenesis strategy [61] with pcDNA3.0-HA-BFRF1 template and the primers specified in Table S1.

### Indirect immunofluorescence

For the detection of HA-BFRF1, Flag-BFLF2, BGLF4, lamin A/C, emerin, TSG101 or Alix, Slide-cultured HeLa cells were transfected with plasmids expressing HA-BFRF1, Flag-BFLF2 or vector pcDNA3.0. The slides were fixed with 4% paraformaldehyde in PBS at 24 h post transfection at RT for 20 min, washed with PBS (145 mM NaCl, 1.56 mM Na_2_HPO_4_, 1 mM KH_2_PO_4_, pH 7.2) and permeabilized with 0.1% Triton X-100 at RT for 5 min. The slides were then incubated with anti-HA (Covance or GeneTex), anti-Flag (Sigma-Aldrich or Viogene), rabbit anti-BGLF4 serum, anti-lamin A/C (Santa Cruz), anti-emerin (Santa Cruz), anti-TSG101 (GeneTex), or rabbit anti-Alix serum [62] at 37°C for 1.5 h. For the detection of major viral capsid components BcLF1, BORF1 or BDLF1, fixed NA cells were permeabilized with 0.1% Triton X-100 and incubated with anti-BcLF1 L2, or rabbit polyclonal anti-BORF1 and anti-BDLF1 antibodies (which were gifts from Dr. Shih-Tung Liu, Chang Gung University, Taiwan), at 37°C for 1.5 h. After washing with PBS for 5 min three times, slides were incubated with Rhodamine-, FITC- or AMCA (amino-methyl-coumarin-acetate)-conjugated anti-mouse or rabbit Ig antibodies (CAPPEL) at 37°C for 1 h. DNA was stained with Hoechst 33258 at RT for 30 sec. The staining patterns were observed under fluorescence or confocal microscopy (Ziess). To observe cell morphology, the margin of cells was detected by MetaMorph software (Molecular Devices) and indicated by white outline in some figures.

### Transmission electron microscopy (TEM) analysis

A total of 3×10^5^ HeLa cells were transfected with plasmid expressing HA-BFRF1 or control vector pcDNA3.0 for 24 h and processed for TEM analysis. Briefly, the cells were trypsinized, pelleted and washed in 0.1 M phosphate buffer (pH 7.4). Pellets were rinsed in 0.1 M phosphate buffer and fixed in 4% paraformaldehyde for 30 min at 4°C. Cells were washed and postfixed in 1% osmium tetroxide for 10 min at room temperature. Sample were dehydrated with increasing concentrations of ethanol from 70 to 100% and then infiltrated with propylene oxide for 1 h, propylene oxide: Epon = 1∶1 for 1 h and then pure Epon for 2 h. Sample were embedded by curing at 40°C for 24 h, followed by 60°C for 48 h prior to sectioning for TEM. Embedded samples were cut into 65-nm-thick sections and stained with uranyl acetate and lead citrate. Samples were imaged using a HITACHI H-7100 transmission electron microscopy, and images were acquired using AMT camera system.

### Co-immunoprecipitation assay

EBV positive NA or HeLa cells (1×10^7^) were transfected with plasmids expressing Rta, HA-BFRF1, Flag-BFLF2, GFP-Chmp4b, Vps4A-DN or relative vector control pSG5, pcDNA 3.0 (Invitrogen), pEGFP-C1 (Clontech) or pDsRed-Mono (Clontech) to match equal total DNA amounts. At 24 h post transfection, cells were harvested and disrupted in RIPA buffer. Cell lysates were centrifuged for 10 min at 16,000× g, to remove the insoluble fraction. Before immunoprecipitation, the lysate was pre-cleared with 125 µl of 20% protein A-Sepharose beads (Pharmacia) for 1 h at 4°C. To immunoprecipitate HA-BFRF1, Flag-BFLF2, TSG101, Alix or GFP-Chmp4b, lysates were incubated with anti-HA (Covance) or anti-Flag (1.5 µg, Sigma-Aldrich), anti-TSG101 (3 µg, GeneTex), anti-GFP antibody (2 µg, Clontech) or rabbit anti-Alix serum (1 µl) at 4°C for 1 h. Protein A-Sepharose beads (125 µl at 20%) were then added to pull down the immunocomplexes with rotation for 1.5 h at 4°C. The immunocomplexes were then washed extensively with RIPA lysis buffer and cold PBS, disrupted in SDS-sample buffer and displayed in 10% SDS-PAGE for immunoblotting.

### Subcellular fractionation of viral DNA and capsid proteins

Subcellular fractionation was basically performed as described previously [63]. Transfected NA cells (5×10^6^) were treated with hypotonic buffer (10 mM Tris, pH8.0, 60 mM KCl, 1.5 mM MgCl_2_, 0.5% NP-40, 1 mM PMSF) on ice for 1 h. After centrifugation at 200× g for 5 min, the supernatant was harvested as the cytosolic fraction. The pelleted nuclear fraction was then washed twice with hypotonic buffer and PBS at 4°C and resuspended separately in DNA extraction buffer (1% SDS, 10 mM Tris-HCl, pH7.6, 10 mM EDTA, 400 mM NaCl, 100 µg/mL RNase A, 200 µg/mL proteinase K) at 55°C overnight or RIPA lysis buffer. For viral genome detection, the viral DNA was further purified by phenol-chloroform extraction and analyzed by qPCR. For protein expression analysis, the total cell lysate, nuclear or cytosolic fraction derived from equal cell numbers was applied to SDS-PAGE for immunoblotting. PARP and α-Tubulin were detected as nuclear and cytosolic markers, respectively.

## Supporting Information

Figure S1 **EBV reactivation redistributes ESCRT components and induces punctate structure formation.** (**A**) EBV-positive NA cells were transfected with plasmid expressing GFP-Chmp4b, GFP (the GFP vector), mCherry-Vps4-DN or DsRed (the mCherry related vector) together with a plasmid expressing Rta or pSG5 vector control. The cells were then harvested and subjected to immunoblotting analysis at 96 h post induction. Protein expression was detected using anti-Zta (4F10), anti-Rta (467), anti-BMRF1 (88A9), anti-BGLF4 (2616), anti-BFRF1 (E10), anti-BFLF2 (C1), anti-BcLF1 (L2), anti-BLLF1 (201), anti-GFP (JL-8), anti-Vps4 (H-165) or anti-lamin A/C in immunoblotting. Lamin A served as a loading control. (**B**) To determine the subcellular localization of Chmp1b in EBV reactivated cells, slide-cultured NA cells were transfected with plasmid expressing Chmp1b-GFP together with Rta expressing or vector plasmid for 24 h, fixed in 4% paraformaldehyde and stained for BFRF1 (red) and DNA. (**C**) To observe Alix, BFRF1 and BFLF2 distribution in Rta expressing cells, slide-cultured NPC-TW01 cells were transfected with Rta expressing or vector plasmid for 48 h. Cells were then fixed and stained for Alix and BFLF2 (green), BFRF1 and Rta (red), and DNA. (**D**) Slide-cultured NPC-TW01 or NA cells were transfected with a plasmid expressing Rta. At 72 h post transfection, cells were fixed and immuno-stained for BFRF1 (red) and BFLF2 (green). BFRF1 and BFLF2 colocalized in cytoplasmic punctate structures.
(TIF)

Figure S2 **The subcellular distribution of EBV BFRF1 and cellular ESCRT components in BFRF1 transfected HeLa cells.** (**A and B**) HeLa cells were transfected with pcDNA3.0 or HA-BFRF1 expressing plasmid together with plasmids expressing organelle markers, YFP-ER for the endoplasmic reticulum, YFP-Golgi for the Golgi apparatus or YFP-Endo for endosomes. At 24 h post transfection, cells were fixed and stained for HA (red in B) and DNA to indicate the organelle distribution in normal (A) or BFRF1-expressing cells (B). (**C**) To observe the subcellular localization of CFP-BFRF1 and GFP-Vps4A, slide-cultured HeLa cells were transfected with plasmids expressing CFP-BFRF1 and wild type GFP-Vps4A for 24 h and stained for DNA. CFP-BFRF1 colocalized predominantly with GFP-Vps4A at the nuclear rim and partially with cytoplasmic vesicles (cyan, Merge). (**D**) Slide-cultured HeLa cells were transfected with plasmids expressing CFP-BFRF1 and mCherry-Vps4-DN for 24 h and stained for DNA. The original color of mCherry (red) was converted to green to facilitate interpretation of the data. Perinuclear CFP-BFRF1/mCherry-Vps4-DN aggregates without cytoplasmic vesicles were observed in transfected cells (cyan, Merge). (**E**) To determine the subcellular distribution of TSG101 in BFRF1-BFLF2 co-expressing cells, HeLa cells were cotransfected with plasmids expressing HA-BFRF1 and Flag-BFLF2. At 24 h post transfection, cells were fixed by 4% paraformaldehyde and immuno-stained for endogenous TSG101 by monoclonal antibody 4A10 or rabbit anti-serum r654 together with HA or Flag.
(TIF)

Figure S3 **The subcellular distribution of HSV-1 UL34 and UL31, and amino acid sequence alignment of EBV BFRF1 homologs.** (**A and B**) For HSV-1 UL34 and UL31 distribution in transiently transfected cells, slide-cultured HeLa cells were transfected with a plasmid expressing HA-UL34 or Flag-UL31 (A), or cotransfected with both plasmids (B). At 24 h post transfection, the cells were fixed with 4% paraformaldehyde, stained for HA (red), Flag (red in A and green in B), and emerin (A, green), and DNA. (**C**) EBV BFRF1 was aligned with herpesviral homologs, including gammaherpesvirus KSHV ORF67, MHV68 (Murine herpesvirus 68) ORF67 and EBV BFRF1, alphaherpesvirus HSV-1 UL34 and PrV (Pseudorabies virus) UL34, and the betaherpesvirus HCMV pUL50 using ClustalW2. Consensus amino acid residues are shown in red, and partial consensus (>50%) residues are shown by blue frames. HCMV pUL50 has a longer carboxyl-terminal region than the other viral homologs. The predicted putative L domains in LD1 and LD2 are indicated by gray boxes. (**D**) Phylogenetic analysis of KSHV ORF67, MHV68 ORF67, EBV BFRF1, HCMV pUL50, HSV-1 UL34 and PrV UL34. PAM (Point Accepted Mutations), 1 unit of evolution as the amount of evolution that will change 1 in 100 amino acids on average.
(TIF)

Figure S4 **EBV BFRF1 redistributes emerin in transfected cell and forms multimers in a native gel.** (**A**) Slide-cultured HeLa cells were transfected with HA-BFRF1 wild type (WT), ΔLD1, ΔLD2, ΔID, ΔESR or ΔTM-expressing plasmids for 24 h, stained for HA (red), emerin (green) and DNA and observed by confocal microscopy. (**B**) Lysates from HeLa cells transfected with vector or plasmid expressing HA-BFRF1 together with GFP-BFRF1 were immunoprecipitated with antibody against HA or GST. The immunocomplexes were then resolved by 10% SDS-PAGE and immunoblotted with antibodies against GFP or HA. The GFP-BFRF1 was coimmunoprecipitated with HA-BFRF1.
(TIF)

Figure S5 **Expression of dominant negative Vps4 or the BFRF1 ΔESR mutant abolishes the formation of punctate structures and redistributes Alix and emerin in cells replicating the virus.** (**A**) EBV-positive NA cells were transfected with vector or plasmid expressing Rta and mCherry-Vps4-DN. At 72 h post transfection, cells were fixed in 4% paraformaldehyde, immuno-stained for BFRF1 distribution (green) in the presence or absence of mCherry-Vps4-DN (red) and observed by confocal microscopy. (**B and C**) To observe the effect of BFRF1ΔESR in cells replicating the virus, slide-cultured NA cells were transfected with plasmid expressing Rta for 48 h followed by transfection of HA-BFRF1 plasmid or HA-BFRF1ΔESR for an additional 24 h. Transfected cells were fixed in 4% paraformaldehyde, immuno-stained for cellular Alix (B, green), emerin (C, green) and HA (red) and observed by confocal microscopy.
(TIF)

Table S1 **Oligonucelotides primers and plasmid DNA templates used in this study.**
(PDF)
