# Supplementary figures and images for: The ESCRT Machinery Is Recruited by the Viral BFRF1 Protein to the Nucleus-Associated Membrane for the Maturation of Epstein-Barr Virus
Source: PLoS Pathog. 2012 Sep 6;8(9):e1002904. doi: 10.1371/journal.ppat.1002904 (PMC3435242; doi:10.1371/journal.ppat.1002904)

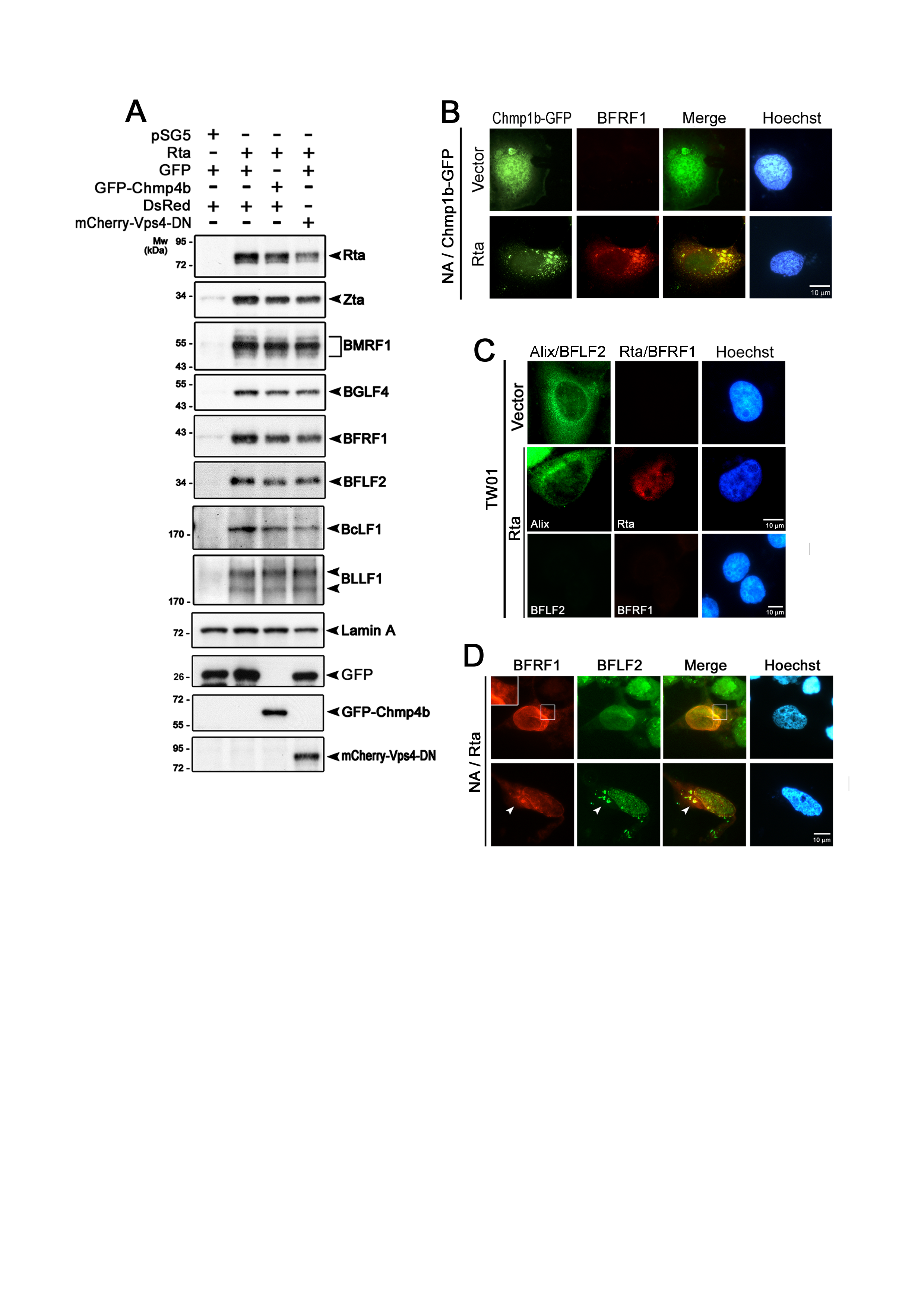

Supplement: Figure S1 — EBV reactivation redistributes ESCRT components and induces punctate structure formation. (A) EBV-positive NA cells were transfected with plasmid expressing GFP-Chmp4b, GFP (the GFP vector), mCherry-Vps4-DN or DsRed (the mCherry related vector) together with a plasmid expressing Rta or pSG5 vector control. The cells were then harvested and subjected to immunoblotting analysis at 96 h post induction. Protein expression was detected using anti-Zta (4F10), anti-Rta (467), anti-BMRF1 (88A9), anti-BGLF4 (2616), anti-BFRF1 (E10), anti-BFLF2 (C1), anti-BcLF1 (L2), anti-BLLF1 (201), anti-GFP (JL-8), anti-Vps4 (H-165) or anti-lamin A/C in immunoblotting. Lamin A served as a loading control. (B) To determine the subcellular localization of Chmp1b in EBV reactivated cells, slide-cultured NA cells were transfected with plasmid expressing Chmp1b-GFP together with Rta expressing or vector plasmid for 24 h, fixed in 4% paraformaldehyde and stained for BFRF1 (red) and DNA. (C) To observe Alix, BFRF1 and BFLF2 distribution in Rta expressing cells, slide-cultured NPC-TW01 cells were transfected with Rta expressing or vector plasmid for 48 h. Cells were then fixed and stained for Alix and BFLF2 (green), BFRF1 and Rta (red), and DNA. (D) Slide-cultured NPC-TW01 or NA cells were transfected with a plasmid expressing Rta. At 72 h post transfection, cells were fixed and immuno-stained for BFRF1 (red) and BFLF2 (green). BFRF1 and BFLF2 colocalized in cytoplasmic punctate structures. (TIF) [file ppat.1002904.s001.tif]

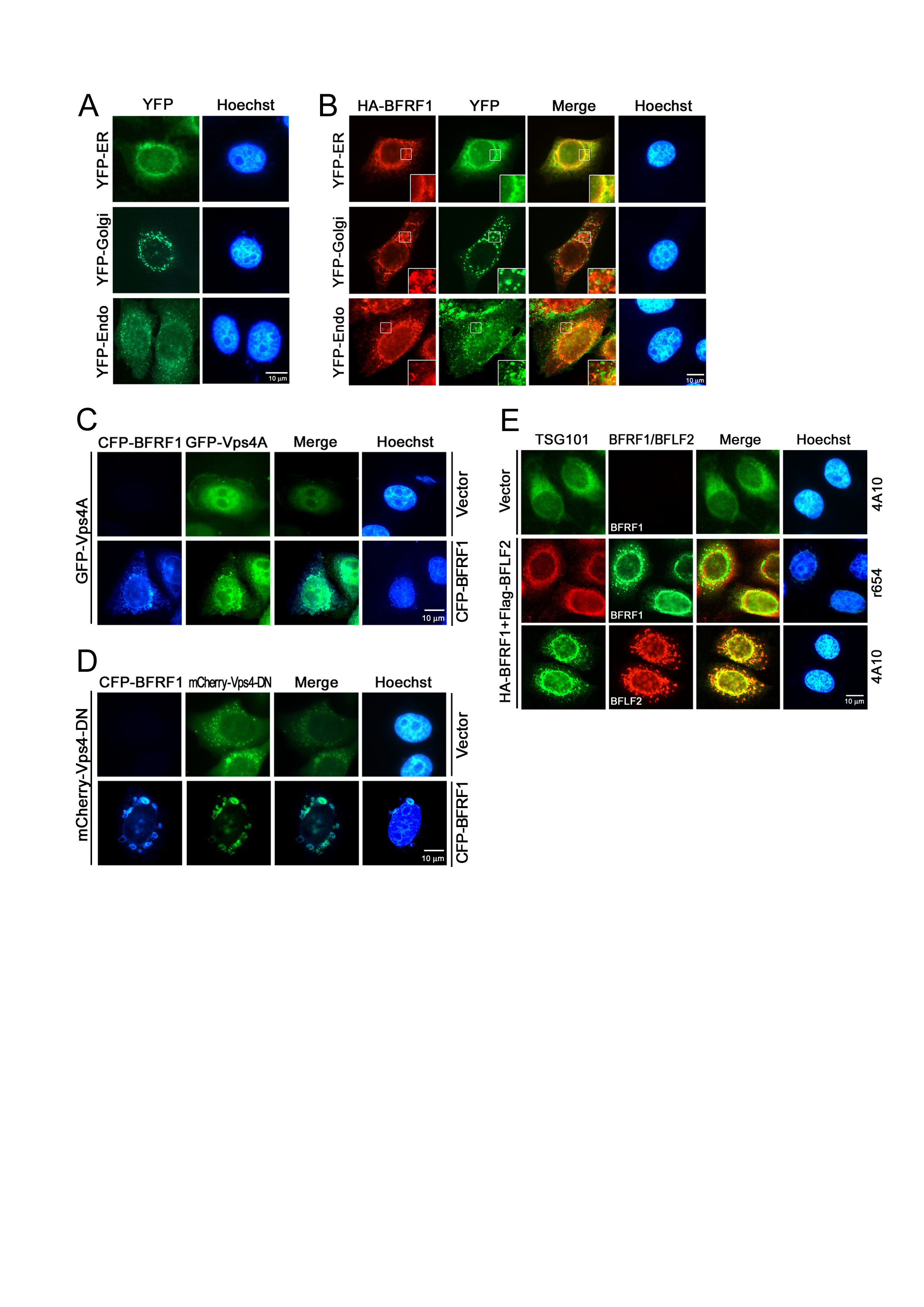

Supplement: Figure S2 — The subcellular distribution of EBV BFRF1 and cellular ESCRT components in BFRF1 transfected HeLa cells. (A and B) HeLa cells were transfected with pcDNA3.0 or HA-BFRF1 expressing plasmid together with plasmids expressing organelle markers, YFP-ER for the endoplasmic reticulum, YFP-Golgi for the Golgi apparatus or YFP-Endo for endosomes. At 24 h post transfection, cells were fixed and stained for HA (red in B) and DNA to indicate the organelle distribution in normal (A) or BFRF1-expressing cells (B). (C) To observe the subcellular localization of CFP-BFRF1 and GFP-Vps4A, slide-cultured HeLa cells were transfected with plasmids expressing CFP-BFRF1 and wild type GFP-Vps4A for 24 h and stained for DNA. CFP-BFRF1 colocalized predominantly with GFP-Vps4A at the nuclear rim and partially with cytoplasmic vesicles (cyan, Merge). (D) Slide-cultured HeLa cells were transfected with plasmids expressing CFP-BFRF1 and mCherry-Vps4-DN for 24 h and stained for DNA. The original color of mCherry (red) was converted to green to facilitate interpretation of the data. Perinuclear CFP-BFRF1/mCherry-Vps4-DN aggregates without cytoplasmic vesicles were observed in transfected cells (cyan, Merge). (E) To determine the subcellular distribution of TSG101 in BFRF1-BFLF2 co-expressing cells, HeLa cells were cotransfected with plasmids expressing HA-BFRF1 and Flag-BFLF2. At 24 h post transfection, cells were fixed by 4% paraformaldehyde and immuno-stained for endogenous TSG101 by monoclonal antibody 4A10 or rabbit anti-serum r654 together with HA or Flag. (TIF) [file ppat.1002904.s002.tif]

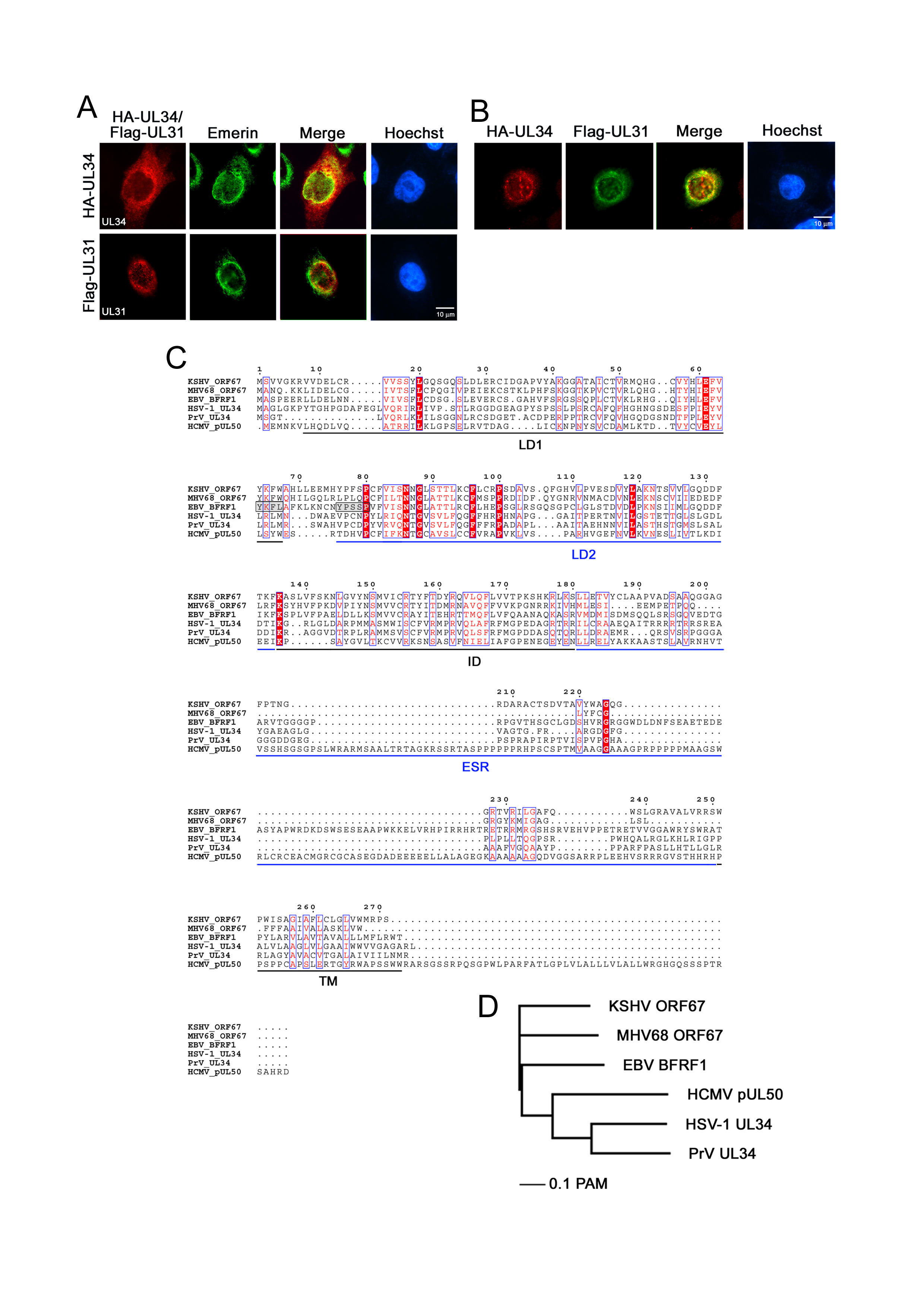

Supplement: Figure S3 — The subcellular distribution of HSV-1 UL34 and UL31, and amino acid sequence alignment of EBV BFRF1 homologs. (A and B) For HSV-1 UL34 and UL31 distribution in transiently transfected cells, slide-cultured HeLa cells were transfected with a plasmid expressing HA-UL34 or Flag-UL31 (A), or cotransfected with both plasmids (B). At 24 h post transfection, the cells were fixed with 4% paraformaldehyde, stained for HA (red), Flag (red in A and green in B), and emerin (A, green), and DNA. (C) EBV BFRF1 was aligned with herpesviral homologs, including gammaherpesvirus KSHV ORF67, MHV68 (Murine herpesvirus 68) ORF67 and EBV BFRF1, alphaherpesvirus HSV-1 UL34 and PrV (Pseudorabies virus) UL34, and the betaherpesvirus HCMV pUL50 using ClustalW2. Consensus amino acid residues are shown in red, and partial consensus (>50%) residues are shown by blue frames. HCMV pUL50 has a longer carboxyl-terminal region than the other viral homologs. The predicted putative L domains in LD1 and LD2 are indicated by gray boxes. (D) Phylogenetic analysis of KSHV ORF67, MHV68 ORF67, EBV BFRF1, HCMV pUL50, HSV-1 UL34 and PrV UL34. PAM (Point Accepted Mutations), 1 unit of evolution as the amount of evolution that will change 1 in 100 amino acids on average. (TIF) [file ppat.1002904.s003.tif]

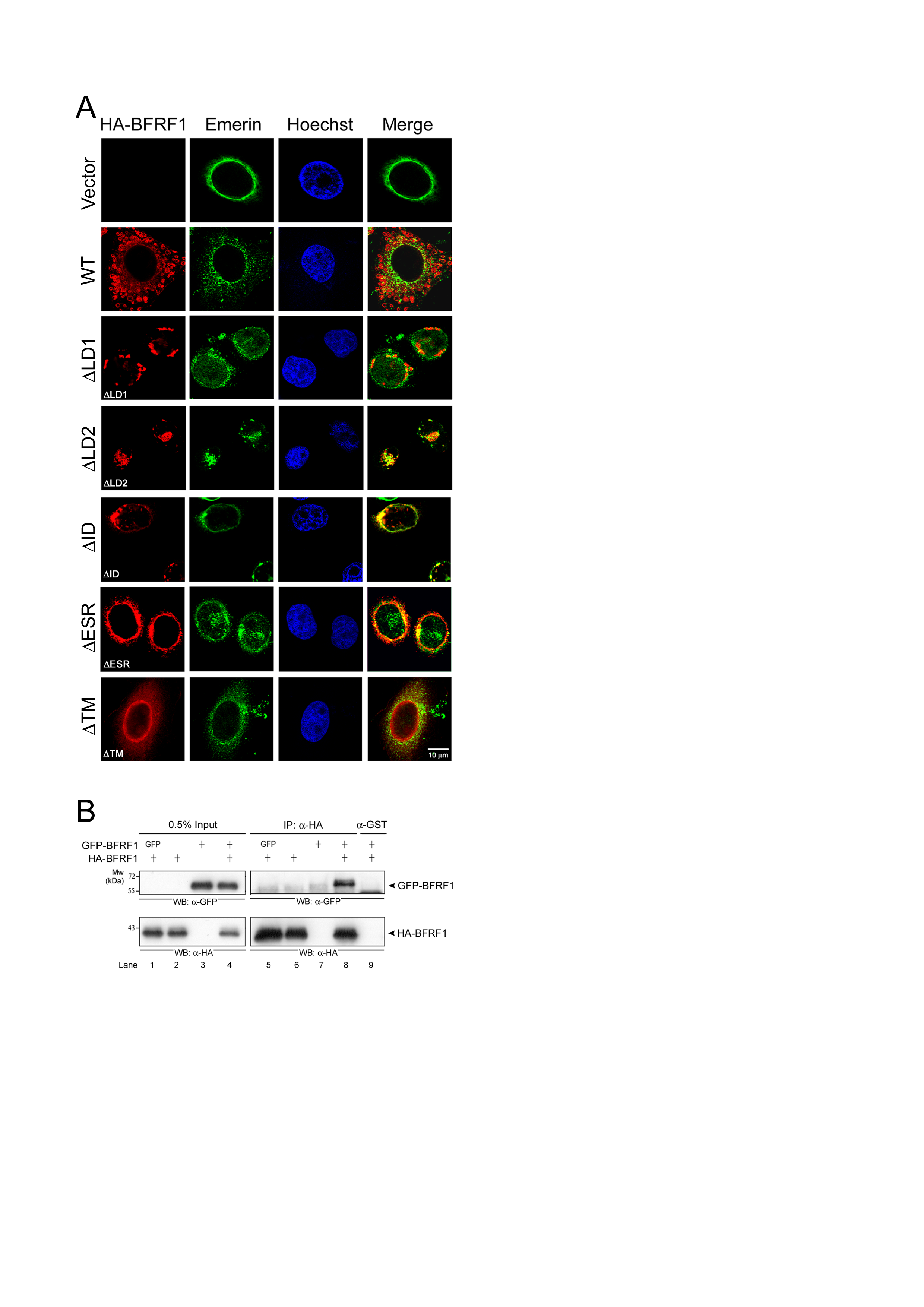

Supplement: Figure S4 — EBV BFRF1 redistributes emerin in transfected cell and forms multimers in a native gel. (A) Slide-cultured HeLa cells were transfected with HA-BFRF1 wild type (WT), ΔLD1, ΔLD2, ΔID, ΔESR or ΔTM-expressing plasmids for 24 h, stained for HA (red), emerin (green) and DNA and observed by confocal microscopy. (B) Lysates from HeLa cells transfected with vector or plasmid expressing HA-BFRF1 together with GFP-BFRF1 were immunoprecipitated with antibody against HA or GST. The immunocomplexes were then resolved by 10% SDS-PAGE and immunoblotted with antibodies against GFP or HA. The GFP-BFRF1 was coimmunoprecipitated with HA-BFRF1. (TIF) [file ppat.1002904.s004.tif]

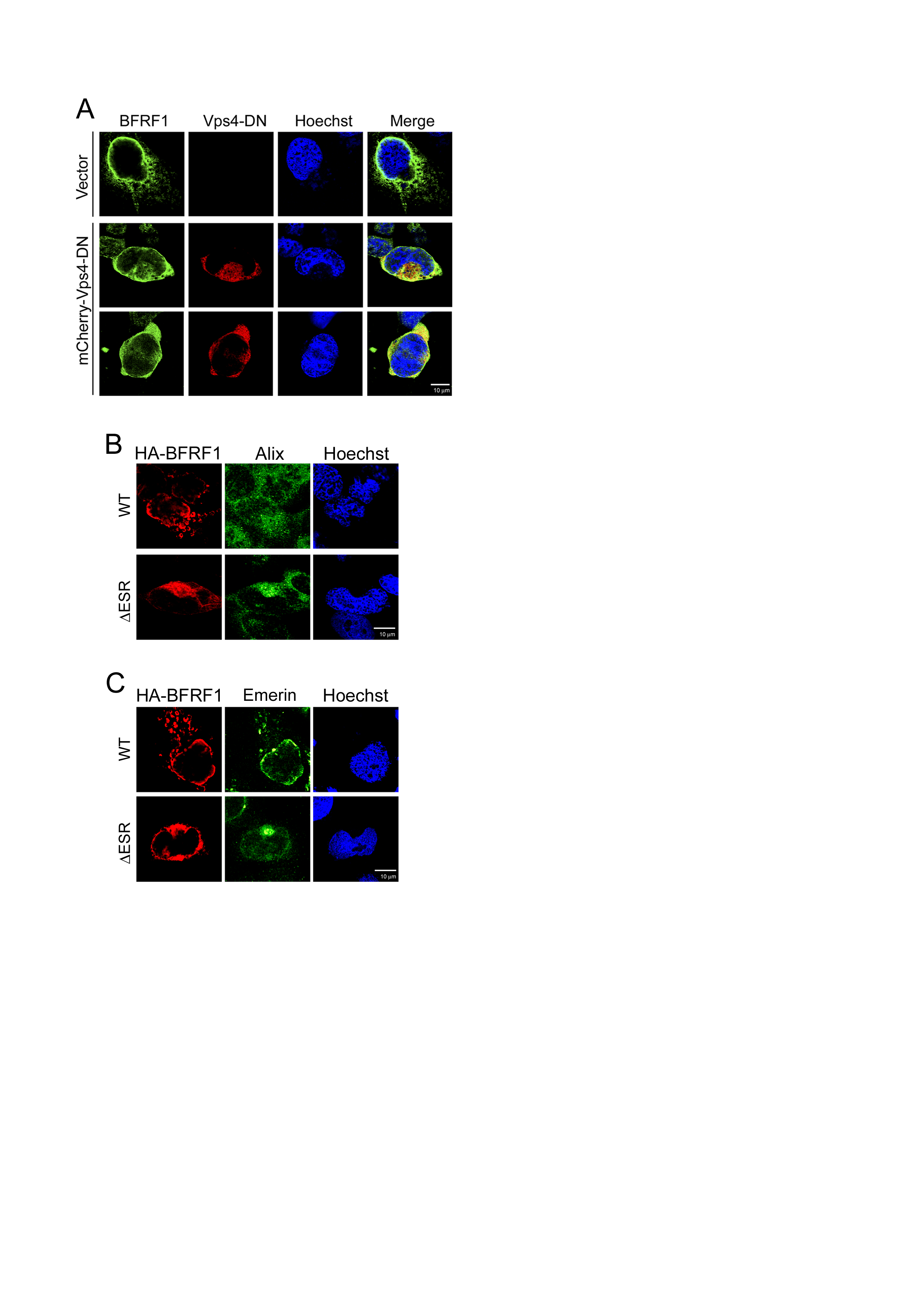

Supplement: Figure S5 — Expression of dominant negative Vps4 or the BFRF1 ΔESR mutant abolishes the formation of punctate structures and redistributes Alix and emerin in cells replicating the virus. (A) EBV-positive NA cells were transfected with vector or plasmid expressing Rta and mCherry-Vps4-DN. At 72 h post transfection, cells were fixed in 4% paraformaldehyde, immuno-stained for BFRF1 distribution (green) in the presence or absence of mCherry-Vps4-DN (red) and observed by confocal microscopy. (B and C) To observe the effect of BFRF1ΔESR in cells replicating the virus, slide-cultured NA cells were transfected with plasmid expressing Rta for 48 h followed by transfection of HA-BFRF1 plasmid or HA-BFRF1ΔESR for an additional 24 h. Transfected cells were fixed in 4% paraformaldehyde, immuno-stained for cellular Alix (B, green), emerin (C, green) and HA (red) and observed by confocal microscopy. (TIF) [file ppat.1002904.s005.tif]
