# Supplementary material for: The ESCRT Machinery Is Recruited by the Viral BFRF1 Protein to the Nucleus-Associated Membrane for the Maturation of Epstein-Barr Virus
Source: PLoS Pathog. 2012 Sep 6;8(9):e1002904. doi: 10.1371/journal.ppat.1002904 (PMC3435242; doi:10.1371/journal.ppat.1002904)
Supplement: Table S1 — Oligonucelotides primers and plasmid DNA templates used in this study. (PDF) [file ppat.1002904.s006.pdf]

Table S1. Oligonucelotides primers and plasmid DNA templates used in this study.

Plasmid	Deleted Region	Primer	Template
HA-BFRF1			
ΔLD1 (pLPT4)	d(8-65)	5'-ATG GCG AGC CCG GAA GAG AGG GCC TTT AAG CTG AAG AAC TGC-3'	pcDNA3.0-HA-BFRFl
ΔLD2 (pLPT5)	d(74-134)	5'-TAA GCT GAA GAA CTG CAA C AAA AGC CCC CTG GTC TTC C-3'	pcDNA3.0-HA-BFRFl
ΔID (pLPT6)	d(135-179)	5'-GCC AGG ATG ACT TCA TTA AGT TC GTC ATG GAT ATG ATT AGT GAT AT-3'	pcDNA3.0-HA-BFRFl
ΔESR (pLPT7)	d(180-313)	5'-ACG CCC AGA AGG CCT CGC GG ACA CCT TAT CTG GCA CGG GT-3'	pcDNA3.0-HA-BFRFl
ΔTM (pLPT8)	d(314-336)	5'-TGG CGT TAT TCT TGG CGC GCC TAG AGG GCC CTA TTC TAT AGT-3'	pcDNA3.0-HA-BFRFl
HSV-1			
HA-UL34 (pLPT23)		Forward 5'-GAT ATC ATG GCG GGA CTG GGC AAG-3'	HSV-1 infected A549 DNA extract
		Reverse 5'-CCG CTC GAG TTA TAG GCG CGC GCC AGC-3'	
3XFlag-UL31 (pLPT24)		Forward 5'-CCC AAG CTT ATG TAT GAC ACC GAC CCC C-3'	HSV-1 infected A549 DNA extract
		Reverse 5'-G GAA TTC CTA CGG CGG AGG AAA CTC G-3'	
